# New Strains of the Deep Branching Streptophyte *Streptofilum*: Phylogenetic Position, Cell Biological and Ecophysiological Traits, and Description of *Streptofilum arcticum* sp. nov

**DOI:** 10.1111/1462-2920.70033

**Published:** 2025-01-08

**Authors:** Karin Glaser, Tatiana Mikhailyuk, Charlotte Permann, Andreas Holzinger, Ulf Karsten

**Affiliations:** ^1^ Institute of Biological Sciences, Biology/Ecology Technical University Bergakademie Freiberg Freiberg Germany; ^2^ M.G. Kholodny Institute of Botany National Academy of Sciences of Ukraine Kyiv Ukraine; ^3^ Department of Botany University of Innsbruck Innsbruck Austria; ^4^ Institute for Biological Sciences, Applied Ecology and Phycology University Rostock Rostock Germany; ^5^ Interdisciplinary Faculty, Department of Maritime Systems University of Rostock Rostock Germany

**Keywords:** cell coverage, desiccation, ecophysiology, ITS2 secondary structure, PI‐curve, *rbc*L phylogeny, SSU rRNA, *Streptofilum*, TEM, temperature

## Abstract

*Streptofilum capillatum* was recently described and immediately caught scientific attention, because it forms a phylogenetically deep branch in the streptophytes and is characterised by a unique cell coverage composed of piliform scales. Its phylogenetic position and taxonomic rank are still controversial discussed. In the present study, we isolated further strains of *Streptofilum* from biocrusts in sand dunes and Arctic tundra soil. Molecular and morphological characterisation including transmission electron microscopy confirmed that both new strains belong to *Streptofilum*. The Arctic strain is described as a new species, *Streptofilum arcticum* sp. nov., based on molecular differences, a specific sarcinoid morphology and unique ultrastructure with massive cell coverage composed of pili‐shaped scales. A comprehensive characterisation of the ecophysiological traits of both new *Streptofilum* isolates and the original one revealed a broad temperature tolerance, a rapid recovery of photosynthetic performance after desiccation, an efficient photosynthesis at low light and a tolerance to high‐light conditions. In addition, *Streptofilum* could cope with UV irradiation, but only *S. capillatum* grew under UV exposure. All *Streptofilum* strains are well‐adapted to water‐deprived terrestrial habitats such as biocrusts. From this study it can be concluded that already early‐branching streptophytes were able to tolerate terrestrial conditions.

## Graphical abstract

The deep branching streptophyte algae *Streptofilum* is characterised by a unique cell coverage. Here, we present new strains of the genus and characterised those strains comprehensively. We found that the unique coverage is a common feature in this genus and described a new species within the genus based on morphological and molecular differences.

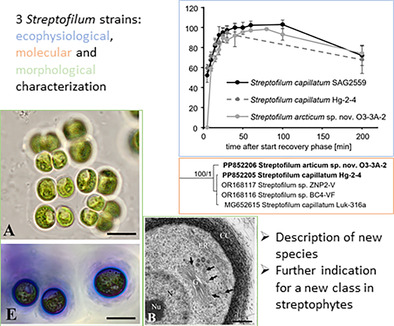

## 1 Introduction

Streptophyte green algae contain the closest living relatives of land plants and thus, have been in research focus for decades as living fossils in the evolutionary process of terrestrialization. A number of fundamental innovations enabled land plants to colonise terrestrial ecosystems, for example, complex cell walls, roots and stomata. However, molecular evidence indicate that streptophyte algae already had key adaptations to terrestrial life long before vascular plants developed (Harholt, Moestrup, and Ulvskov 2016; De Vries and Archibald 2018; Dadras et al. 2023). Multicellularity emerged already in the Klebsormidiophyceae (Bierenbroodspot et al. 2024), and we only start to understand the diversity of the steptophytes most distant to land plants (Irisarri et al. 2021). Streptophyte green algae comprises many aero‐terrestrial taxa with various adaptive traits to survive outside aquatic habitats, such as a self‐protective filamentous lifestyle, excretion of mucilage sheds, accumulation of UV‐sunscreen compounds and potentially flexible cell walls to prevent plasmolysis during desiccation events (Holzinger and Karsten 2013; Herburger and Holzinger 2015; Hartmann et al. 2020).

A new streptophyte genus was discovered just few years ago, *Streptofilum* which exhibited two unique characteristics and thus caught broad attention (Mikhailyuk et al. 2018): first, although the phylogenetic position of this alga could not be clearly solved, it seemed to be a deep‐branching streptophyte. Second, *Streptofilum* is characterised by a unique cell coverage with pili‐shaped scales, and at the TEM level distinct from the scales of *Mesostigma* or *Chlorokybus* zoospores. Those two algal genera are basal streptophytes representing the earliest branches of the phylogenetic tree of streptophyte alga. Other basal streptophytes, like *Klebsormidium* and vegetative cells of *Chlorokybus*, develop a cellulose cell wall, similar to the primary cell wall of plants. In sharp contrast, a cell coverage composed of pili‐shaped structures as observed in *Streptofilum* was never described before.

Recent publications used more complex molecular data and gave an improved insight in the phylogenetic position of *Streptofilum* (Mikhailyuk et al. 2018). One study sequenced the chloroplast genomes of American isolates of *Streptofilum* and several basal streptophytes (Glass et al. 2023). The authors confirmed the first conclusions (Mikhailyuk et al. 2018) that *Streptofilum* represents indeed a deep branch close to the most ancestral streptophyte classes Mesostigmatapyhceae and Chlorokybophyceae and might even be assigned to an own class. Another publication sequenced the transcriptome of the original *Streptofilum* isolate and several strains from Klebsormidiophyceae. Contrary to previous publications, the published phylogenetic tree showed *Streptofilum* within the Klebsormidiophyceae (Bierenbroodspot et al. 2024). A new manuscript, which is not yet peer‐reviewed, evaluated the molecular data of Bierenbroodspot et al. (2024) and indicated contamination in the transcriptome of *Streptofilum*, which could explain the contrasting results on the phylogenetic position of this genus (Žárský and Eliáš 2024). The preliminary phylogenetic results after removing the potential contaminating sequences are in line with Glass et al. (2023) and Mikhailyuk et al. (2018) indicating that *Streptofilum* represents indeed a deep branch among the early‐diverged streptophytes. The ongoing controversial debate clearly points out that there are still uncertainties about the phylogenetic position of *Streptofilum*. Therefore, more research with additional strains are urgently needed to establish a stable phylogenetic positioning of this unique genus.

Recently, two new strains of *Streptofilum* were isolated in biocrusts from deserts in the United States (Glass et al. 2023). Biocrusts can be regarded as a microecosystem with microalgae as primary producers and drivers of biogeochemical activities. As a consequence of microbial activity, biocrust microecosystems can create microclimatic conditions and steep physico‐chemical gradients, which might be less harsh compared to bare soil. For example, the water retention is changed by biocrusts compared to bare soil due to the excretion of extracellular polymeric substances (Chamizo et al. 2016; Geraldes and Pinto 2021). The microclimatic conditions in the biocrusts foster unique microbial assemblages (Glaser et al. 2022). Such moderate microclimatic conditions probably allow various algal taxa to thrive, which might be otherwise rare and less abundant in bare soil, like *Streptofilum*. A previous publication described the original *Streptofilum* strain as adapted to low‐light and less desiccation tolerant compared to other basal streptophytes (Pierangelini et al. 2019).

For this study, we isolated two new *Streptofilum* strains from biocrusts inhabiting coastal sand dunes of the Baltic Sea and polar tundra soils at Spitsbergen. Based on these locations, which differ in their environmental conditions, we hypothesized that the genus *Streptofilum* has the ecophysiological potential to occur in a wide range of habitats from hot and cold deserts to mesic regions. A wide biogeographic distribution would generally require euryoecius adaptive traits, which were experimentally evaluated. We expected that the Artic strain is characterised by a lower optimum growth temperature and higher desiccation tolerance than the strains from temperate regions because of the colder and drier environment in polar regions. Further, we investigated the phylogenetic position by *rbc*L phylogeny, SSU rRNA and ITS2 secondary structure and hypothesized that the unique cell coverage is a common cell biological feature among members of *Streptofilum*.

## 2 Material and Methods

### 2.1 Habitats and Culture Conditions

The original *Streptofilum* strain SAG 2559 was isolated from arable sandy soil in Czech Republic (Mikhailyuk et al. 2018). The new strain Hg‐2‐4 was isolated from a biocrust in coastal sand dunes of the Baltic Sea (Heiligendamm, Germany), details on sampling location were published earlier (Khanipour Roshan et al. 2020). The other new strain O3‐3A‐2 was isolated from a biocrust collected from tundra soil in Spitsbergen, details on sampling location published elsewhere (Kern et al. 2019).

Isolation, purification and establishment of clonal cultures followed the procedure according to Samolov et al. (2020). The cultures were grown in 3 N BBM medium (Starr and Zeikus 1993) at 20°C under low light conditions (~50 μmol photons m^−2^ s^−1^) and a 16:8 h light:dark cycle. Before each experiment, the strains were acclimated to the experimental conditions at least 4 days in advance. The strains were deposited in IBASU‐A, M.G. Kholodny Institute of Botany of NASU of Ukraine, Kyiv, Ukraine under accession numbers IBASU‐A‐780 and IBASU‐A‐781 and are kept as duplicates under the original strain numbers at the University of Rostock.

### 2.2 Light Microscopy

Morphological examinations of both clonal strains were performed at different culture age: 3 weeks, 1–2 months and 1 year with Olympus BX51 and BX53 light microscopes (Olympus, Tokyo, Japan), Nomarski differential interference contrast (DIC) optics and ×40 and ×100 objective lenses. Photomicrographs were taken with digital cameras Olympus UC30 and LC30 (Olympus, Tokyo, Japan). The mucilage cover of the cells was stained with 1% methylene blue. The dimensions of 30 cells were measured; results are given as a range of average value (standard deviation) and in addition the minimum and maximum values in parenthesis.

### 2.3 Histological Observations and Transmission Electron Microscopy (TEM)

For TEM samples were either chemically fixed or high pressure frozen (HPF) followed by freeze substitution (FS) according to published protocols (Aichinger and Lütz‐Meindl 2005; Holzinger, Roleda, and Lütz 2009). Samples were either embedded in Agar Low viscosity resin kit (Agar Scientific, Essex, UK) or modified Spurr's resin (Mikhailyuk et al. 2018). For histological observations, semithin sections (~0.6 μm) were prepared with a Reichert Ultracut (Leica Microsystems, Wien, Austria) and 0.3% Toluidine blue stained sections were viewed at a Zeiss Axiovert 200 M light microscope (Zeiss, Jena, Germany). For TEM, ultrathin sections (~60 nm) were prepared and counterstained with 2% uranyl acetate and Reynold's lead citrate. TEM micrographs were taken on a Zeiss Libra 120 TEM (Carl Zeiss AG, Oberkochen Germany) at 80 kV, equipped with a TRS 2 k SSCCD camera and operated by ImageSP software (Albert Tröndle Restlichtverstärker Systeme, Moorenweis, Germany).

### 2.4 DNA Isolation, Phylogeny and Secondary Structure

Genomic DNA was extracted using the NucleoSpin Plant II mini kit (Macherey Nagel, Düren, Germany). Amplification of SSU rRNA including ITS region and *rbc*L genes followed previously published protocols including calculation of *rbc*L phylogenetic tree (Mikhailyuk et al. 2018). Phylogenetic trees were constructed in the program MrBayes 3.2.2 (Ronquist and Huelsenbeck 2003), using an evolutionary model GTR + G + I, with 5,000,000 generations. Two of the four runs of the Markov chain Monte Carlo were made simultaneously, with the trees taken every 500 generations. Split frequencies between runs at the end of the calculations were below 0.01. The trees selected before the likelihood rate reached saturation were subsequently rejected. The reliability of tree topology was verified by maximum‐likelihood (ML) analysis, using the program GARLI 2.0, and the bootstrap support was calculated with 1000 replicates. The rbcL tree is presented as Figure 5, the SSU phylogenetic tree is presented in Figure S3.

To construct secondary structures of ITS2, the ITS2 sequence was compared with published sequences and secondary structures of other streptophyte algae: *Klebsormidium* (Glaser et al. 2017; Samolov et al. 2019), *Hormidiella parvula, Streptosarcina arenaria* (Mikhailyuk et al. 2018), *Chlorokybus atmophyticus* (Irisarri et al. 2021), *Interfilum paradoxum*, *Entransia fimbriata*, *Spirotaenia condensata* and *Mesostigma viride*. The secondary structures of ITS2 of the last four representatives were built de novo using published sequences (Mikhailyuk et al. 2008; Sluiman, Guihal, and Mudimu 2008; Cheng et al. 2019). Helices were folded with the online software mfold (Zuker 2003) and visualised in the online tool Pseudoviewer (Byun and Han 2009).

### 2.5 Desiccation Experiment

The experiment followed the procedure published earlier (Karsten, Herburger, and Holzinger 2016) with following variation: 100 mL LiCl solution (40% w/v) was filled in each chamber to achieve desiccating conditions in the air‐tight chamber, which resulted in stable relative humidity (RH) of around 47% (MSR 145 W; MSR Electronics GmbH, Switzerland). *Streptofilum* biomass was transferred onto a glass fibre filters, and positioned in the desiccation chamber (four replicates). The yield of photosystem II (YII) was measured as a proxy for the vitality of the cells using non‐invasive pulse amplitude modulation fluorometry (PAM2500, Walz, Germany). The signal was recorded during the desiccation process every 30 min for up to 4.5 h. After YII signals completely declined, the filters were rewetted with 250 μL medium (3 N BBM, see culture conditions), transferred to a chamber filled with 100 mL water (RH ~95%) and recovery was monitored. YII was recorded every 5–10 min for 1.5 h and additionally after 24 h.

### 2.6 Photosynthesis‐Irradiance (PI) Curves

PI curves of the three *Streptofilum* strains (four replicates per strain) were measured according to the protocol published earlier (Prelle et al. 2019). Briefly, ~3 mL of thin log phase algal suspension of each strain and 2 mM final concentrationof NaHCO_3_ were added to four airtight water‐tempered (20°C) oxygen electrode chambers (DW1, Hansatech Instruments, King's Lynn, UK). The oxygen concentration was measured at 10 increasing photon flux density levels ranging from 0 to ~1.500 μmol photons m^−2^ s^−1^ of photosynthetically active radiation (PAR), using a non‐invasive oxygen dipping probe (DP sensors PreSens Precision Sensing GmbH, Regensburg, Germany). Chlorophyll *a* (Chl *a*) was extracted after the experiment (using 96% ethanol at 70°C for 10 min) and quantified spectrophotometrically (Ritchie 2006).

### 2.7 Temperature‐Dependent Oxygen Production and Consumption

The photosynthetic and respiratory responses of each *Streptofilum* strain (*n* = 4) at temperatures between 5°C and 40°C were measured using the same oxygen optode system as for the PI curves. After 20 min incubation in the dark, the respiratory oxygen consumption (10 min in the dark), followed by the photosynthetic oxygen production (10 min under light‐saturated conditions at 335 μmol photons m^−2^ s^−1^ PAR) were determined. Measurements were normalised to the Chl *a* concentration (see procedure above).

### 2.8 Growth Rate Under UV Exposure

The fluorescence of Chl *a* was used as a proxy for biomass to calculate the temperature‐dependent growth rates of the three *Streptofilum* strains. The in vivo Chl *a* fluorescence measurements were performed according to the previously published protocol (Karsten, Klimant, and Holst 1996). The cultures were grown in disposable plastic Petri dishes, kept at constant temperature of 20°C, and measured every 24 h for 4 days. In addition to PAR, UV emitting light bulbs were used. The Petri dishes were covered with two different foils: one foil allowed the UV radiation to pass, the second foil absorbed light below 400 nm (control) (Kitzing, Pröschold, and Karsten 2014). The UV‐treatment was undertaken in triplicates. Both treatments were exposed to 80–90 μmol photons m^−2^ s^−1^ (Lumilux Deluxe Daylight L15W/950; OSRAM), the UV‐treated cells were additionally exposed to 6–7 W m^−2^ s^−1^ UV‐A and 0.37–0.45 W m^−2^ s^−1^ UV‐B (Q‐Panel‐UVA 340 fluorescent lamps, Cleveland, USA).

### 2.9 Statistical Analyses

All statistical analyses were done in R, version 4.2.1 (R Development Core Team 2022) or Microsoft Excel. Photosynthetic irradiance (PI) curves were fitted using the Walsby model in Excel, based on least‐square method (Walsby 1997). Based on this model, maximum rates of net primary production (NPP_max_), respiration (*R*), light utilisation coefficient (*α*), photoinhibition coefficient (*β*), light saturation point (*I*_k_), and the light compensation point (*I*_c_) were calculated. Temperature curves were fitted in R using the model published by Yan and Hunt, also based on the least‐square method (Yan and Hunt 1999). Confidence intervals for maximum oxygen production, optimum and maximum temperatures were calculated using the command ‘confint2’ (package nlstools).

## 3 Results

### 3.1 Morphological Characterisation of Two New *Streptofilum* Strains

**FIGURE 1:**
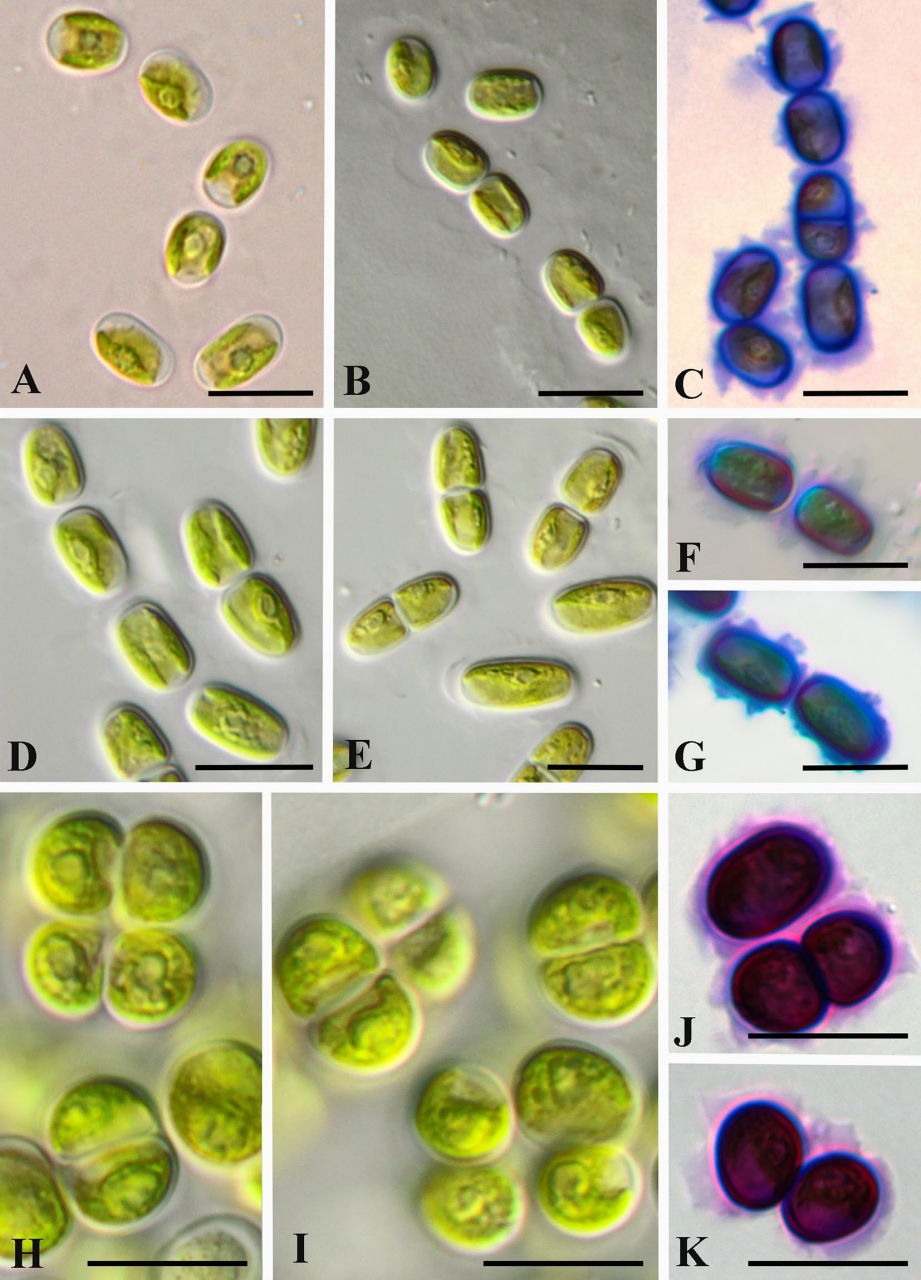
Micrographs of *Streptofilum* strains: General view of filaments or packets surrounded by mucilage envelope (A, B, D, E, H, and I) and staining of mucilage with Methylene blue (C, F, G, J, and K); (A–C) *Streptofilum capillatum* SAG 2559, (D–G) *Streptofilum capillatum* Hg‐2‐4, (H–K) *Streptofilum arcticum* sp. nov. O3‐3A‐2. Scale bars: 10 μm.

Strain Hg‐2‐4 (IBASU‐A‐780) was morphologically similar to the authentic *Streptofilum capillatum* (SAG 2559, IBASU‐A‐521, Figure 1A–C). Cells were grouped in short filamentous‐like structures, often disintegrated to diads and unicells, and surrounded by homogenous mucilage with waved or lobbed edge (Figure 1D–G). Hg‐2‐4 formed smooth colonies on the agar surface. Vegetative cells were ellipsoid to ovoid, (6.1)7.0–8.8(12.1) μm length, and (4.6)5.0–5.8 μm width. Chloroplast was parietal, plate‐shaped with smooth margin and single pyrenoid surrounded by several starch grains. Vegetative reproduction of Hg‐2‐4 occurred by cell division in one plane (sporulation‐like type) with formation of cell diads. Sexual reproduction was not observed. This strain differed from SAG 2559 by slightly longer cells. Both strains were isolated from rather sandy substrates of similar geographical region (Western Europe: Czech Republic and Germany).

**FIGURE 2:**
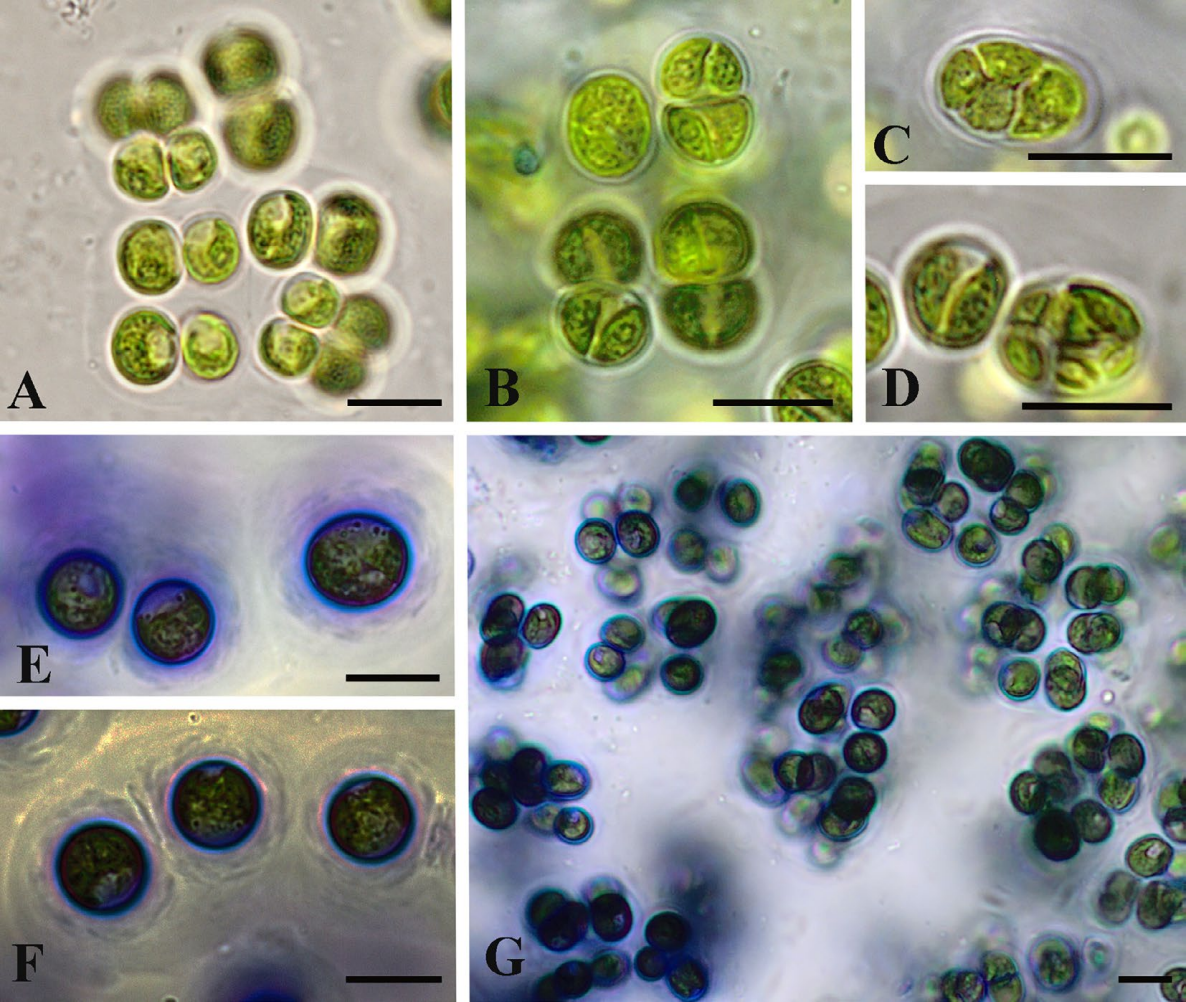
*Streptofilum arcticum* sp. nov. O3‐3A‐2: Cell packet and aggregations surrounded by mucilage (A); reproduction by cell division on 2–4 and 8 daughter cells (B–D); thick layered and finely structured mucilage in old cultures (E, F); general view Radiococcaceae‐like colony with grouped cells surrounded by common mucilage (G); staining of mucilage with Methylene blue (E–G). Scale bars: 10 μm.

Strain O3‐3A‐2 (IBASU‐A‐781) was characterised by a different morphology. It had a sarcinoid thallus, forming 2–4 celled packet‐like and rarely short filamentous‐like aggregations, often disintegrated to diads and unicells (Figures 1H–K and 2A). Cells were surrounded by thick (to 5.0–10.0 μm) layered mucilage with waved edge. Mucilaginous caps on cells and layered finely structured mucilage were especially prominent in old cultures (Figure 2E,F). This alga formed large mucilaginous colonies which resemble Radiococcaceae‐like morphology (Figure 2G). Due to the similar cell morphology (see below) it also resembled a *Chlorokybus*‐like morphology, but with much smaller cells. O3‐3A‐2 formed cluster‐like mucilaginous colonies on the agar surface. Vegetative cells were widely ellipsoid to almost spherical, (5.1)6.4–7.6(11.0) μm in length, and (4.2)–5.5–6.9(8.6) μm in width. Chloroplasts were parietal, plate‐shaped, with a smooth or waved margin and a single pyrenoid surrounded by several starch grains. Vegetative reproduction occurred by cell division in several planes (sporulation‐like type) and formation of sporangia with 2–4 (8) cells (Figure 2B–D). Due to widening of the sporangial cell wall the adult cells were organised in 2–4 celled groups and formed large mucilaginous colonies. Sexual reproduction was not observed. The strain O3‐3A‐2 differed from SAG 2559 by general sarcinoid and packet‐like morphology, much thicker and layered structured mucilage as well as a different shape of the cells. Because of more rounded cells of the strain O3‐3A‐2 cell division took place in several planes that leading to cell packet formation. Strong mucilage prevented disintegration of cell packets after division and promoted the general sarcinoid morphology of this alga.

### 3.2 Ultrastructural Characterisation of *Streptofilum* Shows Unique Cell Coverage With Pili‐Shaped Scales

**FIGURE 3:**
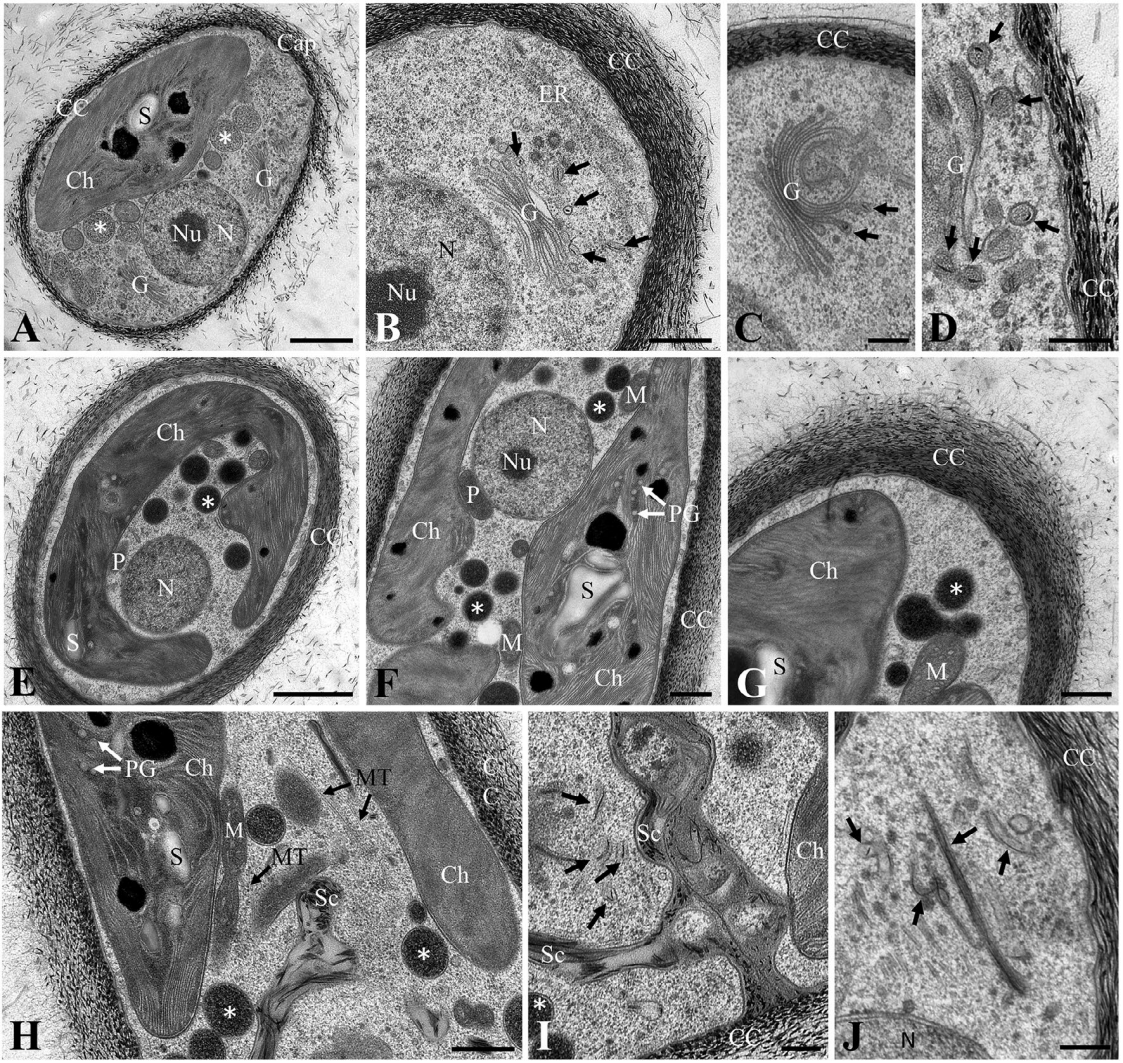
Transmission electron micrographs of *Streptofilum capillatum* SAG 2559 (A–D) and Hg‐2‐4 (E–J) fixed by high pressure freeze fixation. An overview showing cell coverage composed of densely arranged piliform scales close to the plasma membrane and a cap‐area with looser arranged scales, chloroplast with starch grains, membrane‐surrounded bodies with granular content (asterisks) and Golgi bodies, (B) detail with nucleus and nucleolus, Golgi body with several vesicles containing scales (arrows), (C) detail with Golgi body and vesicles containing scales detached from the trans‐side. (D) numerous vesicles containing scales (arrows) close to the plasma membrane, (E) overview of cross section with nucleus, chloroplast and peroxisome, membrane surrounded‐bodies with granular content appear electron dense (asterisk), (F) longitudinal section with parietal chloroplast surrounding the nucleus, one peroxisome, mitochondria, (G) detail view of the cell coverage, scales arranged denser close to plasma membrane, scales more scatted in the mucilage at the outside, (H) longitudinal section during cell division with loosely arranged scales in the newly formed cross wall, microtubules perpendicular to cross wall, (I) detail view of newly formed cross wall, numerous vesicles containing scales (arrows) close to the plasma membrane, (J) longitudinal section of vesicles containing scales (arrows). CC, cell coverage; Ch, chloroplast; G, Golgi body; M, mitochondrion; MT, microtubules; N, nucleus; Nu, nucleolus; P, peroxisome; PG, plastoglobuli; S, starch grain; Sc, scale. Asterisks indicate membrane‐surrounded bodies with granular content, arrows indicate vesicles containing scales. Scale bars: A, E: 1 μm, B, F, G, H: 500 nm; C, D, I, J: 250 nm.

When investigated by TEM after high pressure freeze fixation, *S. capillatum* SAG 2559 (Figure 3A–D), exhibited a cell coverage composed of characteristic electron dense piliform scales. These scales were densely arranged close to the plasma membrane and forming a looser arrangement outside, sometimes forming a cap‐like region (Figure 3A). The cells contained a nucleus with a distinct nucleolus, one chloroplast with starch grains, mitochondria, numerous membrane‐surrounded bodies up to 500 nm in diameter with a granular medium electron dense content and Golgi bodies (Figure 3A). The Golgi bodies formed vesicles containing individual scales (Figure 3B–D), the vesicles were detached at the trans‐side of the Golgi body (Figure 3C), and several vesicles with scales were found close to the plasma membrane (Figure 3D). In high pressure frozen *Streptofilum* strain Hg‐2‐4 a similar arrangement of the cell coverage was observed (Figure 3E–J). In cross‐sections, the parietal arrangement of the chloroplast was visible (Figure 3E). One peroxisome was observed between the nucleus and the chloroplast that contained numerous starch grains and plastoglobules (Figure 3E,F). The membrane‐surrounded bodies with granular contents were electron denser (Figure 3E–I) as in *S. capillatum* SAG 2559. During cell division newly formed cross walls contained a loose arrangement of scales (Figure 3H,I), microtubles were visible perpendicular to the cross walls (Figure 3H). Particularly in the area of the newly formed cross walls many vesicles containing individual scales were observed in the cytoplasm (Figure 3I). When the scale containing vesicles were longitudinally sectioned, scale lengths of up to ~500 nm were observed (Figure 3J).

**FIGURE 4:**
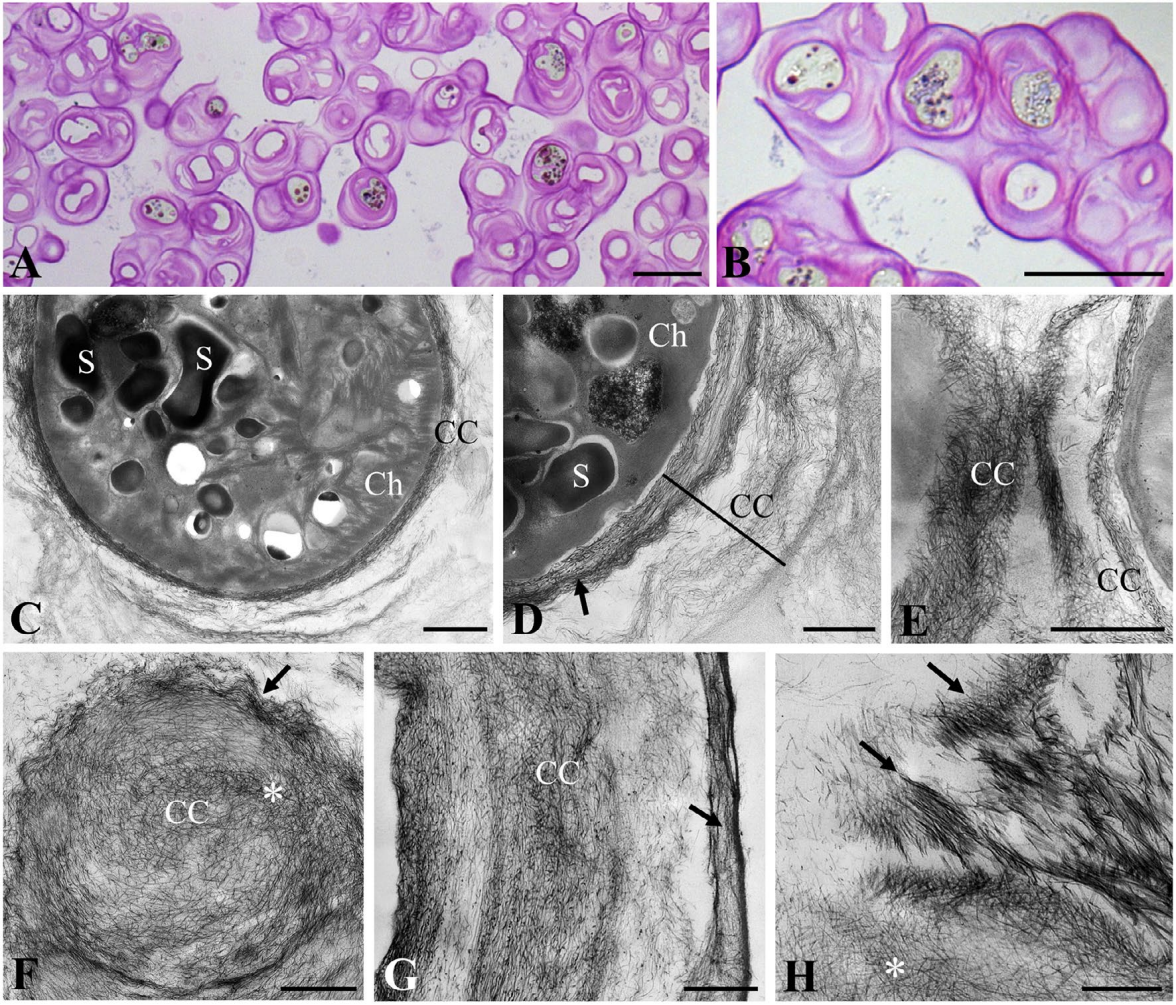
Histological and ultrastructural characterisation of the newly described *Streptofilum arcticum* sp. nov. (O3‐3A‐2). (A, B) Toluidine blue stained semi‐thin sections showing massive multi‐layered cell coverage, (C) cell overview with chloroplast and pyrenoid surrounded by starch grains, cell coverage multilayered, (D) starch grains in chloroplast, arrangement of the scales in the cell coverage is denser (arrow) close to the plasma membrane, multilayered and looser distant from the cytoplasm, (E) connection zone between two cells, (F) tangential surface section showing the net‐like arrangement of the scales in the center (asterisk), and wave like at the surface (arrow), (G) cross section through multi‐layered cell coverage, arrangement of scales with different densities, note the very dense arrangement at the outer surface (arrow), (H) close‐up of scales with changing orientation from net‐like (asterisk) to parallel (arrows). CC, cell coverage; Ch, chloroplast; S, starch grain. Scale bars: A, B: 20 μm; C–F, 1 μm; G, H: 500 nm.

The ultrastructure of strain O3‐3A‐2 (Figure 4) was distinct from both *S. capillatum* strains (see above). Toluidine blue stained semi‐thin sections of chemically fixed material showed sarcinoid cell packets with a massive multilayered cell coverage with a diameter of up to ~5 μm (Figure 4A,B). When viewed by TEM (Figure 4C–H), the cytoplasm had a dense appearance with clearly visible thylakoid membranes in the chloroplast and the pyrenoid was surrounded by starch grains (Figure 4C,D). The piliform scale arrangement of the cell coverage was denser close to the plasma membrane, and looser in more distant position (Figure 4C,D). At the junction of two cells, the piliform scales appeared denser (Figure 4E). Tangential sections showed a net‐like arrangement of the scales (Figure 4F). The multi‐layered arrangement of the scales is illustrated in Figure 4G, where the outermost layer was again much denser when compared to the inner layers. In a close‐up the scales appeared to change orientation from net like to parallel depending on the plain of section (Figure 4H).

### 3.3 Molecular Phylogeny and ITS2 Secondary Structure of *Streptofilum*

**FIGURE 5:**
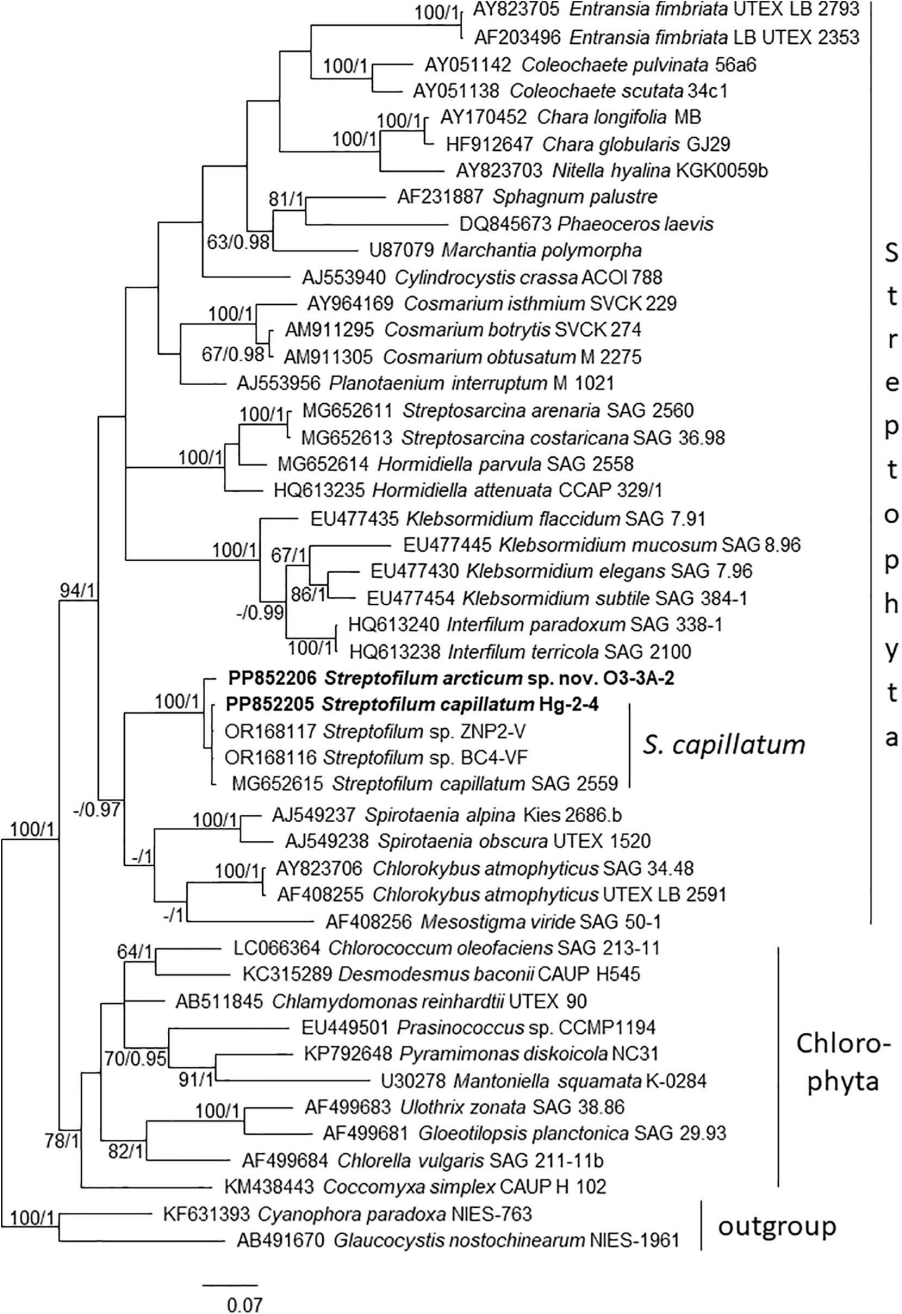
Molecular phylogeny of Streptophyta (and some Chlorophyta species) based on *rbc*L sequence comparisons. A phylogenetic tree was inferred by Bayesian method (program MrBayes) with Bayesian Posterior Probabilities (PP) and maximum likelihood (ML) bootstrap support (BP) indicated at nodes. From left to right, support values correspond to Maximum‐Likelihood BP and Bayesian PP; BP values lower than 60% and PP lower than 0.9 not shown. Strain in bold represents newly sequenced *Streptofilum* strains.

The SSU rRNA sequences of the strains SAG 2559 and Hg‐2‐4 were nearly identical (99.9% identical), similarity of SAG 2559 to O3‐3A‐2 was lower (99.2%, Table 1). Additionally, the partial *rbc*L sequence of SAG 2559 and Hg‐2‐4 were nearly identical (99.8%), but differed to the sequences of O3‐3A‐2 (97.7% identical to SAG 2559). The *rbc*L phylogenetic tree showed all three plus two American *Streptofilum* strains close to each other (Figure 5). The authentic *S. capillatum* strain SAG2559 is very closely positioned to the two American strains (ZNP2‐V and BC4‐VF) and our new strain from the German coastal dunes (Hg‐2‐4). The Arctic strain O3‐3‐2A was found to be located within the *Streptofilum* clade, but separate from the other strains. The lineage *Streptofilum* fell outside of the Klebsormidiophyceae and close to early‐branching Streptophytes.

**TABLE 1:** Sequence identity of three *Streptofilum* strains; upper right the identity of 18S rRNA sequences (bold), down left of *rbc*L sequences (italic).

	SAG 2559	Hg‐2‐4	O3‐3A‐2
SAG 2559		**0.999**	**0.992**
HG‐2‐4	*0.998*		**0.993**
O3‐3A‐2	*0.977*	*0.978*	

**FIGURE 6:**
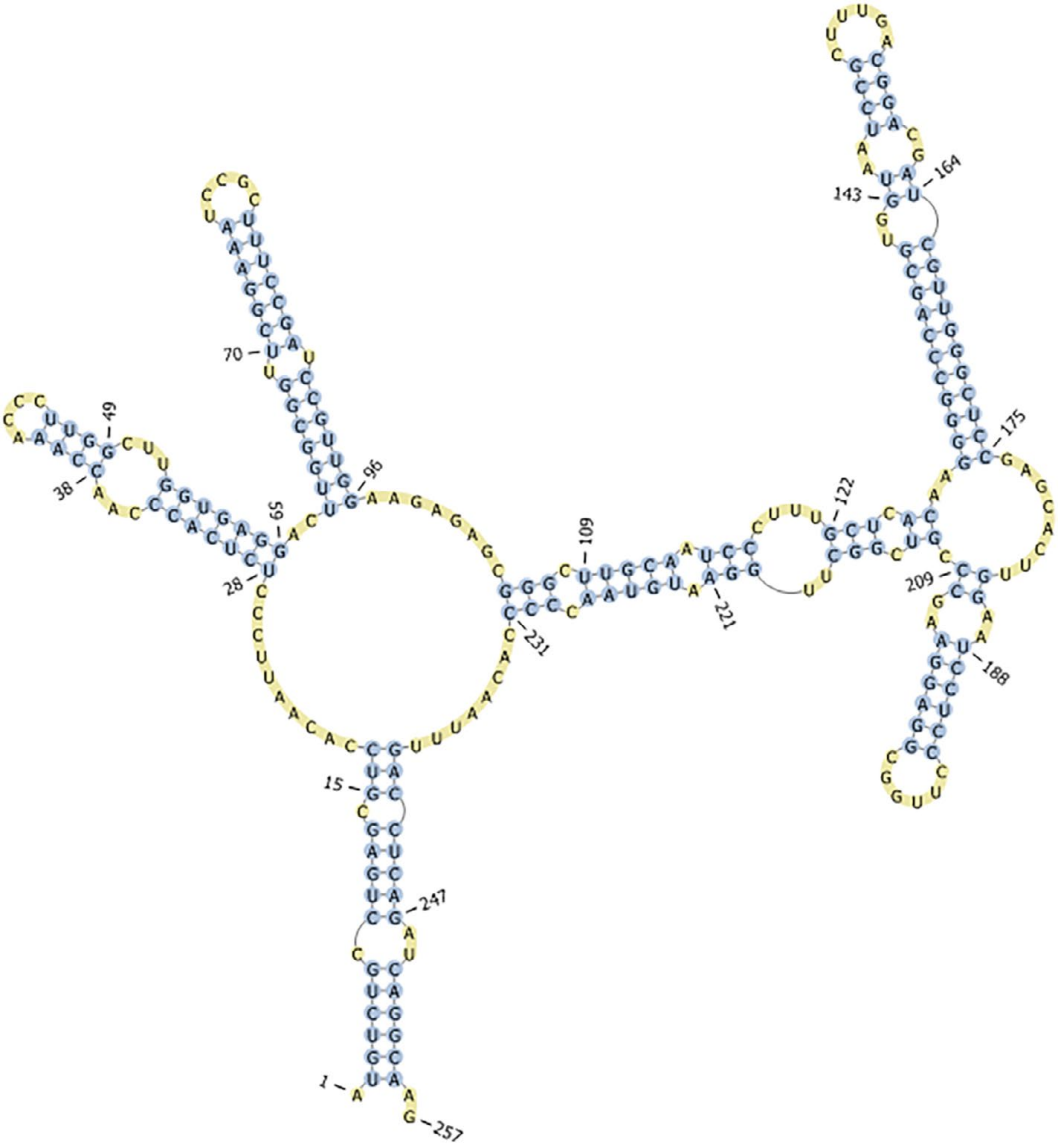
Secondary structure of ITS2 from *Streptofilum arcticum* sp. nov. O3‐3A‐2; pyrimidine‐pyrimidine‐mismatch (= typical for eukaryotic algae) is indicated by the red arrow (GenBank accession number: PP849119).

The ITS2 region of *Streptofilum* strain O3‐3‐2A differed largely from all published sequences: a direct sequence comparison did not result in any hit in the GenBank database. Thus, the secondary structure of the ITS2 region was used for further comparison. The ITS2 secondary structure of the *Streptofilum* strain O3‐3A‐2 was characterised by three helices, of which the third was branched (Figure 6). The first and also the second helix of *Streptofilum* O3‐3A‐2 showed similar characteristic features like other streptophyte and also chlorophyte algae. For example, the pyrimidine–pyrimidine‐mismatch in the second helix is common for all eukaryotes (Caisová, Marin, and Melkonian 2013). *Mesostigma* and *Spirotaenia* have also a branched third helix; but in those two genera a fourth helix is present (Figure S1). *Chlorokybus* is characterised by three helices, but without branches (Irisarri et al. 2021). Representatives of Klebsormidiophyceae (*Klebsormidium, Interfilum, Hormidiella, Streptosarcina* and *Entransia*) were characterised by completely different secondary structure of ITS2 (Figure S2). Their ITS2 was quite uniform in all genera with four helices without branches (Glaser et al. 2017; Mikhailyuk et al. 2018; Samolov et al. 2019).

### 3.4 *Streptofilum* Exhibits Desiccation Tolerance

**FIGURE 7:**
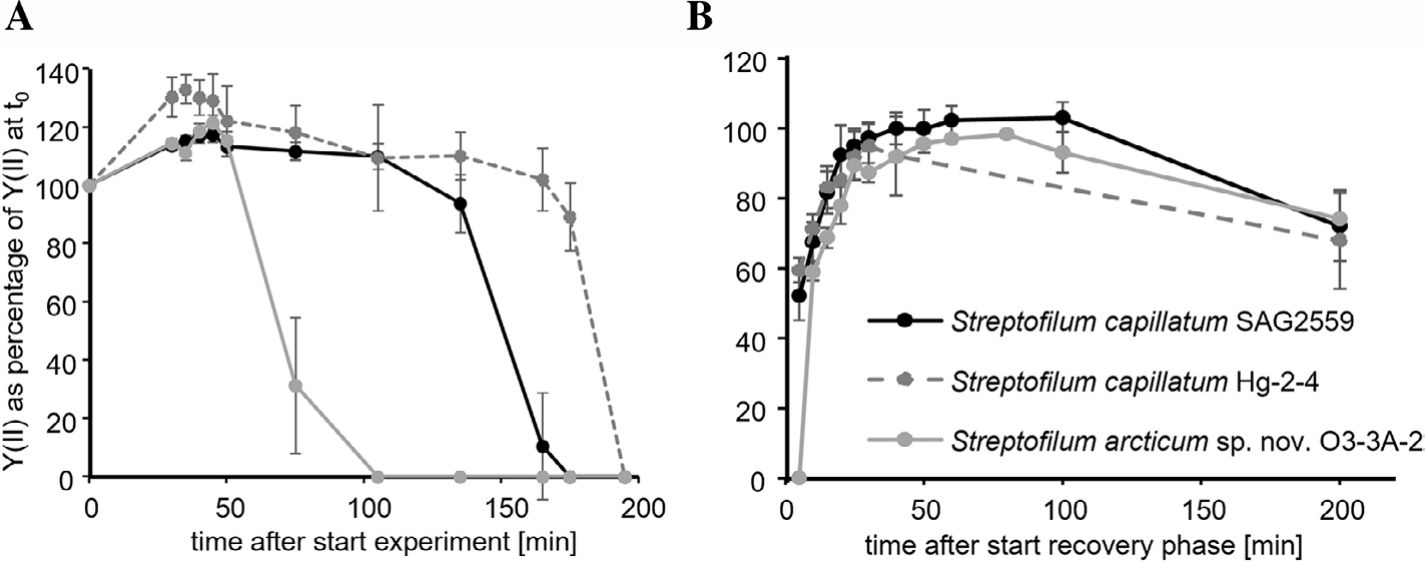
Effect of controlled desiccation (A) and rehydration (B) on the effective quantum yield (Y(II)) of PSII of three *Streptofilum* strains (*n* = 4). Effective quantum yield values were standardised to the starting Y(II) to 100% for better comparison.

The Arctic strain O3‐3‐2A showed no photosynthetic activity after ~110 min of controlled desiccation (at 47% RH), whereas the other two strains sustained around 1 h longer under these conditions (Figure 7A). The kinetics of recovery after rewetting the filters and transferring them to 95% RH was similar for all three strains: directly after rewetting nearly 100% of the initial value was reached, which decreased to ~80% of the initial value after 24 h (Figure 7B).

### 3.5 *Streptofilum* Reveals Low Light Compensation and no Photoinhibition at High Light

**FIGURE 8:**
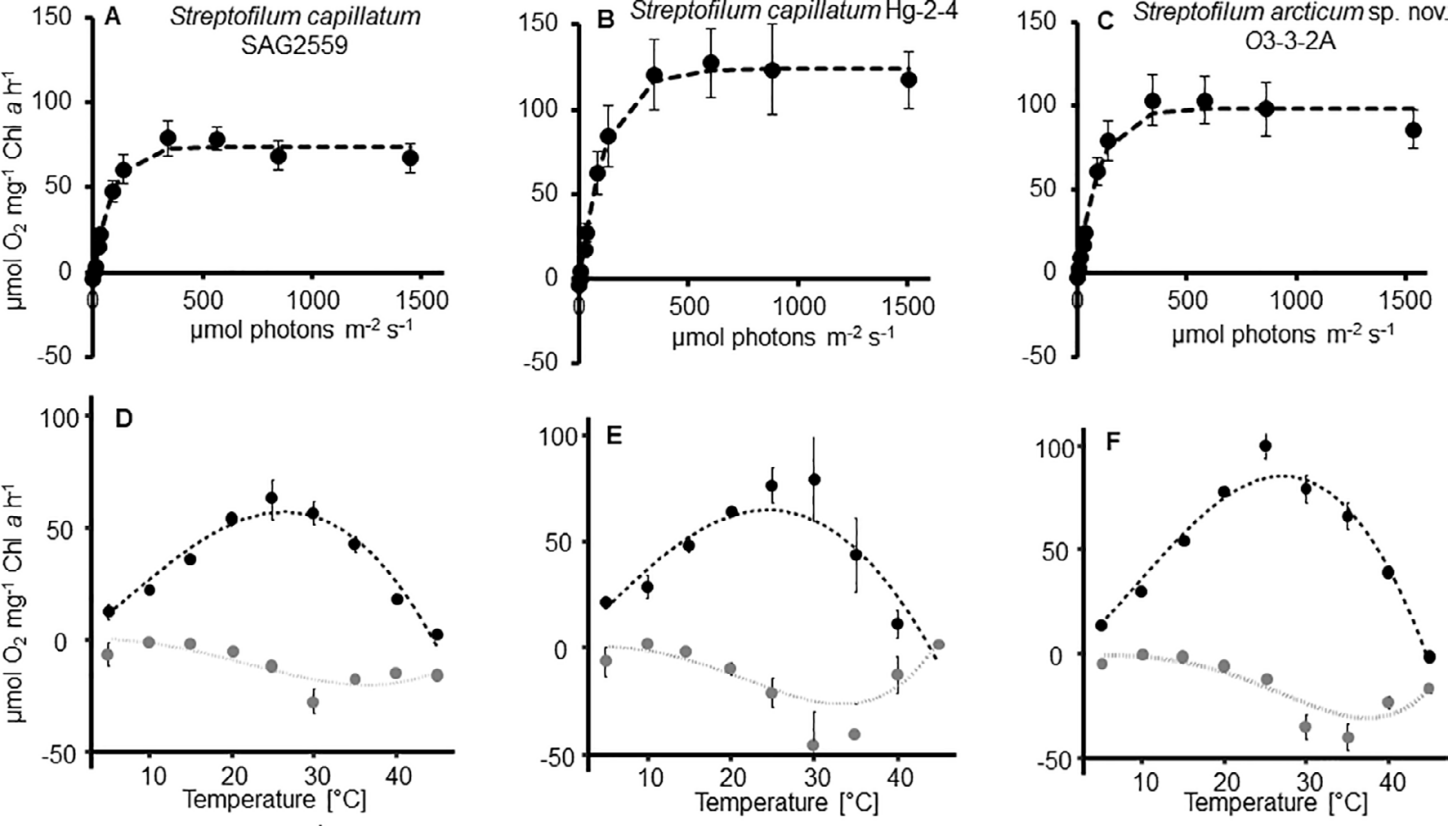
Photosynthetic‐irradiance curve of three *Streptofilum* strains (A–C). The points represent the mean of the measured values (*n* = 4 ± standard deviation), and the dotted line is the fitting curve after Walsby (1997). Parameters of the PI curve are given in Table 2. Temperature‐dependent oxygen production in three *Streptofilum* strains (D–F). The points represent the mean of the measured values (*n* = 4 ± standard deviation), grey colour represents the respiration in the dark, black the photosynthesis. Dotted lines are the fitting curves after Yan and Hunt (1999). (A, D) *Streptofilum capillatum* SAG 2559, (B, E) *Streptofilum capillatum* Hg‐2‐4, (C, F) *Streptofilum arcticum* sp. nov. O3‐3A‐2.

The kinetics of the PI curves was found similar in all three *Streptofilum* strains (Figure 8A–C). No photoinhibition was detected and the light‐compensation point was already reached below ~5 μmol photons m^−2^ s^−1^ (Table 2). Respiration rate, alpha and light saturation point were similar in all isolates (Table 2). In contrast, the maximum photosynthetic oxygen production was different among the strains: SAG 2559 had the lowest net oxygen production of ~78 μmol O_2_ mg^−1^ Chl *a* h^−1^ and Hg‐2‐4 the highest oxygen production of ~127 μmol O_2_ mg^−1^ Chl *a* h^−1^.

**TABLE 2:** Parameters of PI‐curve (Figure 8) for three *Streptofilum* strains (±standard deviation).

	SAG 2559	Hg‐2‐4	O3‐3A‐2
NPP_max_	78.1 ± 5.37	127 ± 19.97	105.1 ± 13.36
*α*	0.89 ± 0.13	1.01 ± 0.13	1.02 ± 0.14
*I*_k_	91.4 ± 6.96	129.3 ± 4.64	105.9 ± 3.95
*I*_c_	4.27 ± 0.78	3.51 ± 0.81	2.89 ± 1.14
Respiration	−3.68 ± 0.6	−3.46 ± 1.16	−2.85 ± 0.82

Abbreviations: *α*, light utilisation coefficient; *l*_c_, light compensation point, respiration is given in μmol O_2_ mg^−1^ Chl *a* h^−1^; *l*_k_, light saturation point; *p*_max_, maximum oxygen production in μmol O_2_ mg^−1^ Chl *a* h^−1^.

### 3.6 Temperature Dependent Photosynthesis and Respiration

All three strains exhibited a broad temperature tolerance between 5°C and 40°C following classical Gauss‐dynamics (Figure 8D–F and Table 3). The optimum temperature for photosynthesis was with ~26°C similar for all three strains, as well as the maximum temperature with ~44°C. The tolerance width for optimum photosynthesis (80% of maximum capacity) was broad, ranging from around 15°C to 35°C (Figure S4). The optimum respiration was measured at 34°C–37°C, which is higher than the respective photosynthesis.

**TABLE 3:** Parameters of oxygen production or consumption along a temperature gradient after fitting with Yan and Hunt model (Figure 8) including 5% confidence interval for three *Streptofilum* strains.

	SAG 2559	Hg‐2‐4	O3‐3A‐2
*p*_max_	55.6 (51.1–60.1)	67.8 (57.6–77.9)	87 (82–92.1)
Optimum temperature P (°C)	26.5 (25–27)	25.3 (22.4–28.2)	27.3 (26.2–28.3)
Maximum temperature P (°C)	44.4 (43.3–45.5)	43.8 (41.8–45.7)	44.5 (43.8–45.3)
Respiration max	−20.6 (−15.96 to −25.14)	−36.5 (−47 to −26.1)	−34.8 (−29.1 to −40.5)
Optimum temperature resp. (°C)	36.7 (33.5–39.8)	33.9 (30.6–37.1)	37 (35.5–38.6)
Maximum temperature resp. (°C)	51 (44–58.1)	44.5 (42.7–46.2)	47.1 (45.3–48.9)

Abbreviations: *p*_max_, maximum oxygen production in μmol O_2_ mg^−1^ Chl *a* h^−1^; respiration max, maximum oxygen consumption in μmol O_2_ mg^−1^ Chl *a* h^−1^.

### 3.7 Differential UV‐Tolerance in the Investigated *Streptofilum* Strains

The *Streptofilum* strains SAG 2559 and Hg‐2‐4 were tolerant to UV treatment as reflected by similar growth rates with and without UV radiation (0.25–0.3 μ day^−1^). In contrast, the Arctic strain O3‐3‐2A surprisingly did not grow under UV radiation. It is worth to mention, that the measured Chl *a* signal remained unchanged over the experiment course (4 days) and the biomass still appeared green after the experiment.

## 4 Discussion

In the present study, a comprehensive characterisation of two newly isolated *Streptofilum* strains in comparison to the authentic *S. capillatum* strain (Mikhailyuk et al. 2018) was performed. While strain Hg‐2‐4 was morphologically very similar, strain O3‐3A‐2 exhibited morphological and some physiological differences. After the first record of *S. capillatum* SAG 2559 (Mikhailyuk et al. 2018), more *Streptofilum* strains were isolated from biocrusts: two strains from (semi‐)arid regions in the United States (Glass et al. 2023) and two strains presented in this study from mesic and Arctic regions. One of the new strains (Hg‐2‐4) was morphologically similar to the authentic strain of *S. capillatum* with very small differences (slightly longer and wider cells of Hg‐2‐4 [average size 7.0–8.8 × 5.0–5.8 μm opposite 5.7–6.7 × 4.6–5.0 μm in SAG 2559]). In contrast, the Arctic strain O3‐3A‐2 differs from the authentic *S. capillatum* by several morphological characters, like cell shape, thallus organisation and mucilage structure. *Streptofilum* is characterised by a unique and outstanding cell coverage (Mikhailyuk et al. 2018). The cell coverage of strain O3‐3A‐2 was composed of the same piliform scales, but they expanded much further in the broad mucilage layers covering the sarcinoid cell packets formed by this strain (Figure 4A–H). Thus, it is likely that the formation of piliform scales is a stable trait and all members of *Streptofilum* have this kind of cell coverage. Some other early‐branching streptophytes are also covered by organic scales: *Mesostigma* and *Chlorokybus* (Rogers, Mattox, and Stewart 1980; Domozych, Wells, and Shaw 1991). Moreover, zoospores and gametes of *Chaetosphaeridium*, *Coleochaete* (both belong to the class Coleochaetophyceae), Charophyceae and even some embryophytes (e.g., *Lycopodium*, *Psilotum*) are covered by submicroscopic organic scales (Moestrup 1970; Maden, Renzaglia, and Whittier 1996; van den Hoek, Mann, and Jahns 1996; Duncan, Renzaglia, and Garbary 1997; Renzaglia et al. 2001). However, the structure and organisation of the cell coverage in *Streptofilum* are distinct from the other mentioned Streptophyta and Embryophyta, the latter characterised by scales of variable complex structures and refined filigree shape clearly organised like fish scales.

### 4.1 Unique ITS2 Secondary Structure Underpins Distinct Phylogenetic Position of *Streptofilum*

The rbcL phylogenetic tree shows *Streptofilum* in one cluster with *Chlorokybus*, *Mesostigma*, *Spirotaenia* and other early‐diverged streptophytes algae; but distant from Klebsomidiophyceae and Phragmoplastophyta. Although this is only a one gene phylogeny, the tree is in line with phylogenetic tree based on whole chloroplast sequencing, which shows *Streptofilum* in‐between *Chlorokybus* and Klebsormidiophyceae (Glass et al. 2023). In order to gain additional insight in the phylogenetic position of *Streptofilum*, we estimated the secondary structure of the ITS2 region and compared its overall topology with other streptophyte lineages. The resulting ITS2 secondary structure of *Streptofilum* has common but also distinguishing features to other streptophyte algae. Members of the class Klebsormidiophyceae are characterised by an ITS2 secondary structure with four helices and an unbranched third helix (Glaser et al. 2017; Mikhailyuk et al. 2018; Samolov et al. 2019). The more basal streptophytes *Mesostigma* and *Spirotaenia* show a secondary structure with four helices and a branched third helix (Figure S1). *Chlorokybus*, in contrast, only has three helices and the third is unbranched (Irisarri et al. 2021). The ITS2 secondary structure of *Streptofilum* combined features of those streptophytes in a unique way: it is characterised by three helices and the third helix is branched. Consequently, the secondary structure of the ITS2 supports the findings by previous studies (see Section 1) that the genus *Streptofilum* represents indeed an independent lineage among the streptophytes.

### 4.2 Description of *Streptofilum arcticum* sp. nov

The molecular analyses and morphological features confirmed that the new strains (Hg‐2‐4 and O3‐3‐2A) also belong to the genus *Streptofilum*. The strain Hg‐2‐4 as well as both American strains (ZNP2‐V and BC4‐VF) were identified as *S. capillatum* , because of the morphological (only for Hg2‐4 available) and molecular similarity to the authentic strain *S. capillatum* SAG2559. However, the Arctic strain O3‐3A‐2 exhibits differences in SSU rRNA and *rbc*L sequences as well as a specific morphology with massive cell coverage containing pili‐shaped scales, and it was found in a special habitat and locality. Therefore, the description of a new species of the monotypic genus *Streptofilum* is proposed.

*Streptofilum arcticum* Mikhailyuk, Glaser, Holzinger et Karsten sp. nov. (Figures 1H–K, 2, and 4).

Description: Thallus sarcinoid, forming 2–4 packet‐like and rarely short filamentous‐like aggregations, often disintegrated to diads and unicells. Cells naked, surrounded by variably dense layers of piliform scales, visible in TEM micrographs possibly of organic nature and thick (to 5.0–10.0 μm) layered mucilage with waved edge. Mucilaginous caps on cells and layered finely structured mucilage are especially prominent in old cultures. Mucilaginous colonies resemble Radiococcaceae‐like general morphology. Vegetative cells widely ellipsoid to almost spherical, (5.1)6.4–7.6(11.0) μm in length and (4.2)–5.5–6.9(8.6) μm in width. Chloroplast parietal, plate‐shaped, with smooth or waved margin and a single pyrenoid surrounded by several starch grains. Vegetative reproduction by cell division in several planes (sporulation‐like type) and formation of sporangia with 2–4 (8) cells. Sexual reproduction not observed.

Habitat: biological soil crusts, Arctic tundra top soil.

Type locality: vicinities of the Ny‐Ålesund Research Station (Svalbard, Norway), Ossian‐Sarsfjellet, tundra top soil, biological soil crusts, 78.95238° N, 12.49632° E.

Holotype (designated here): KW‐A‐32535, preserved culture material of authentic strain O3‐3A‐2 (IBASU‐A‐781), Algotheca, Herbarium of the M.G. Kholodny Institute of Botany of the National Academy of Sciences of Ukraine (KW).

Isotype (designated here in support of the holotype): Preserved specimen 240,521 fixed for TEM, resin embedded material of strain O3‐3A‐2 is available for reference at the Department of Botany, University of Innsbruck, Austria.

Iconotype (designated here in support of the holotype): Figures 1H–K, 2 and 4A–H.

Authentic strain: O3‐3A‐2 was deposited in IBASU‐A, M.G. Kholodny Institute of Botany of NASU of Ukraine, Kyiv, Ukraine, under number IBASU‐A‐781.

Etymology: arcticum = from Latin word *arcticum*—Аrctic.

### 4.3 Ecophysiological Performance Indicates Adaptation to Terrestrial Habitats

The desiccation tolerance of *Streptofilum* spp. was evaluated at 47% RH, where it lost its photosynthetic activity within ~2 h, but fully recovered after rewetting within few minutes, and could maintain ~80% of its initial value after 24 h. Previous experiments revealed that *S. capillatum* SAG 2559 could not recover under harsher conditions at 10% RH (Pierangelini et al. 2019). The latter results could be confirmed for both new *Streptofilum* strains, which did not recover photosynthesis after rapid desiccation at ~10% RH (data not shown). Other terrestrial algae, like *Klebsormidium* and its relative *Entransia* and *Hormidiella*, could recover at least to 50% of initial performance even under extreme and rather unnatural conditions of around 10% RH (Herburger, Karsten, and Holzinger 2016; Donner et al. 2017; Pierangelini et al. 2019). *Streptofilum* cell aggregates easily disintegrate into single cells, which are more prone to desiccation than filaments or cell packets, which characterises *Klebsormidium* and *Entransia*. This could be one reason, why *Streptofilum* could not recover after harsh desiccation (Holzinger and Karsten 2013). In mesic and Arctic regions, the natural habitats of the analysed *Streptofilum* strains, the RH drops down to 10% only rarely and never in such rapid way like under experimental conditions when directly exposed to silica gel (Pierangelini et al. 2019). Thus, the milder desiccating conditions at 47% RH better reflect natural conditions and both *Streptofilum* species proved desiccation tolerant with a fast and nearly full recovery of photosynthetic activity. The geographic origin of the *Streptofilum* strains was not reflected in the respective desiccation tolerance. Although Spitzbergen is characterised by a lower annual precipitation than Central Europe, it exhibits drastic changes in the environmental conditions because of climate change. Due to the influence of the Gulf Stream and the so‐called Arctic amplification particularly western Spitzbergen experiences higher summer and winter temperatures then in previous decades, about 409 mm annual precipitation, more melt‐water from glaciers and more rain‐on‐snow events in winter (Pedersen et al. 2022). All these climatic and hydrological changes not only lead to more water availability in the tundra over the course of the seasons, thereby supporting the so‐called Arctic greening (Pedersen et al. 2022), but also might explain the less‐pronounced desiccation tolerance of the Arctic *Streptofilum* species. Additionally, the biocrust microecosystem, where *Streptofilum* strains Hg‐2‐4 and O3‐3A‐2 were isolated, provides in general less harsh environmental conditions compared to bare soil: in terms of desiccation, the consortium of biocrust organisms accumulate extrapolymeric substances and hence a water‐holding and protective matrix for all cells (Colica et al. 2014; Belnap, Weber, and Büdel 2016).

Both *Streptofilum* species are well adapted to low‐light conditions indicated by the low light compensation point and the high alpha value. On the other hand, *Streptofilum* did not show any indication for photoinhibition even at high light conditions. Adaption to low‐light and tolerance of high‐light conditions was already described for other streptophyte algae and interpreted as high photophysiological plasticity (Karsten, Herburger, and Holzinger 2016; Pierangelini et al. 2017). Biocrusts represent a three‐dimensional micro‐ecosystem with steep vertical light gradients because of shading effects by lichen and moss thalli, algal filaments or aggregates, mucilage, soil particles and so on. The high photophysiological plasticity of *Streptofilum* allows growth at different vertical positions in the biocrust, that is, exposure to high or low light conditions, which might represent a competitive advantage compared to other algal species.

Most aero‐terrestrial algal species are eurytherm and thus, the broad temperature tolerance of all three *Streptofilum* strains is in line with previous reports of other biocrusts algae (Donner et al. 2017; Glaser et al. 2023). In terrestrial habitats, like soil surface or biocrusts, the environmental conditions can change more drastic and faster than in aquatic ecosystems, and consequently, broad temperature tolerance is required to thrive in those habitats. The geographic origin of the *Streptofilum* strains was not reflected by the respective temperature tolerance, all strains exhibited similar optima and ranges. This is confirmed by data on other microalgal species which also showed similar temperature tolerance ranges independent of the geographic origin (Teoh, Phang, and Chu 2013; Borchhardt and Gründling‐Pfaff 2020).

Under experimental UV exposure, both *S. capillatum* strains thrived, but *S. arcticum* sp. nov. did not grow although it survived the treatment. A previous publication indicated that *Streptofilum* might be able to produce mycosporine‐like amino acid (MAAs), known UV sunscreen compounds (Pierangelini et al. 2019). MAAs are wide‐spread in marine and terrestrial algae, including basal streptophytes, but missing in Embryophytes (Hotter et al. 2018; Geraldes and Pinto 2021). The streptophyte *Klebsormidium* synthesises and accumulates various MAAs under UV exposure (Kitzing, Pröschold, and Karsten 2014; Hartmann et al. 2020). Future experiments will clarify, if *S. capillatum* is capable to produce MAAs under UV exposure and if this trait is missing in *S. arcticum* sp. nov., which would explain the lack of growth under UV treatment.

## 5 Conclusion

*Streptofilum capillatum* and the newly described strain *S. arcticum* sp. nov. are early‐diverged streptophyte algae characterised by a unique cell coverage. Three strains of *Streptofilum* were investigated within this study regarding their morphology, ultrastructure, molecular taxonomy and various ecophysiological traits, like photosynthetic performance under light gradient, desiccation and temperature tolerance. The strain Hg‐2‐4 isolated from coastal sand dunes was unambiguously identified as *S. capillatum* . The Arctic strain O3‐3A‐2 differed in SSU rRNA and *rbc*L sequences as well as some morphological features from the authentic *S. capillatum* strain and hence was described as a new species, *S. arcticum* sp. nov. The data on ultrastructure, molecular phylogeny including the secondary structure of ITS2 region pointed towards a separate lineage formed by *Streptofilum*, which is located among other early‐diverged lineages (*Mesostigma*, *Chlorokybus*) in Streptophyta phylogeny. The ecophysiological experiments revealed that *Streptofilum* is well adapted to its terrestrial habitat as it is eurytherm, desiccation‐tolerant and photophysiologically highly plastic. All these traits guarantee ecological success and survival in biocrusts, and we assume that *Streptofilum* has a much broader biogeographic distribution than reported so far. In addition, this study demonstrates that new species and even new lineages can still be found even in common environments. In general, the discovery of new species contributes to broaden our knowledge on biodiversity, and holds the potential for new biotechnological innovations regarding, for example, the presence of secondary metabolites. Such compounds like UV absorbing sunscreens could be used as environmentally friendly sun protection for human skins.

## Author Contributions

**Karin Glaser:** investigation, writing – original draft, visualization, conceptualization, methodology, data curation. **Tatiana Mikhailyuk:** writing – review and editing, methodology, visualization, data curation, investigation, funding acquisition, conceptualization. **Charlotte Permann:** methodology, writing – review and editing, investigation. **Andreas Holzinger:** writing – review and editing, methodology, investigation, funding acquisition, resources. **Ulf Karsten:** funding acquisition, writing – review and editing, conceptualization, resources.

## Conflicts of Interest

The authors declare no conflicts of interest.

## Supporting information


**Figure S1.** ITS2 secondary structures of various Streptophyta, representing different phylogenetic lineages and *Streptofilum*.
**Figure S2.** ITS2 secondary structures of different representatives of Klebsormidiophyceae (Streptophyta).
**Figure S3.** Molecular phylogeny of Streptophyta based on SSU sequence comparisons. A phylogenetic tree was inferred by Bayesian method (program MrBayes) with Bayesian Posterior Probabilities (PP) indicated at nodes; values lower than 0.95 are not shown. Strain in bold represents newly sequenced *Streptofilum* strains.
**Figure S4.** Optimum and maximum temperatures based on short‐term oxygen production of measurements and long‐term (few days, measured as growth‐rate) effects of temperature treatment on three *Streptofilum* strains (*n* = 4). Values were calculated based on the fitting results presented in Figure 8.

## Data Availability

Sequences were deposited at GenBank under the accession numbers PP852206 and PP852205 for *rbc*L, PP844619 and PP844620 for SSU rRNA and PP849119 for ITS2.

## References

[emi70033-bib-0001] Aichinger, N. , and U. Lütz‐Meindl . 2005. “Organelle Interactions and Possible Degradation Pathways Visualized in High‐Pressure Frozen Algal Cells.” Journal of Microscopy 219, no. 2: 86–94. 10.1111/j.1365-2818.2005.01496.x. 16159344

[emi70033-bib-0002] Belnap, J. , B. Weber , and B. Büdel . 2016. “Biological Soil Crusts as an Organizing Principle in Drylands.” In Biological Soil Crusts: An Organizing Principle in Drylands, edited by B. Weber , B. Büdel , and J. Belnap , 3–13. Cham: Springer International Publishing. 10.1007/978-3-319-30214-0_1.

[emi70033-bib-0003] Bierenbroodspot, M. J. , T. Darienko , S. de Vries , et al. 2024. “Phylogenomic Insights Into the First Multicellular Streptophyte.” Current Biology 34, no. 3: 670–681.e7. 10.1016/j.cub.2023.12.070. 38244543 PMC10849092

[emi70033-bib-0004] Borchhardt, N. , and S. Gründling‐Pfaff . 2020. “Ecophysiological Response Against Temperature in Klebsormidium (Streptophyta) Strains Isolated From Biological Soil Crusts of Arctic and Antarctica Indicate Survival During Global Warming.” Frontiers in Ecology and Evolution 8: 153. 10.3389/fevo.2020.00153.

[emi70033-bib-0005] Byun, Y. , and K. Han . 2009. “PseudoViewer3: Generating Planar Drawings of Large‐Scale RNA Structures With Pseudoknots.” Bioinformatics 25, no. 11: 1435–1437. 10.1093/bioinformatics/btp252. 19369500

[emi70033-bib-0006] Caisová, L. , B. Marin , and M. Melkonian . 2013. “A Consensus Secondary Structure of ITS2 in the Chlorophyta Identified by Phylogenetic Reconstruction.” Protist 164, no. 4: 482–496. 10.1016/j.protis.2013.04.005. 23770573

[emi70033-bib-0007] Chamizo, S. , J. Belnap , D. J. Eldridge , Y. Cantón , and O. Malam Issa . 2016. “The Role of Biocrusts in Arid Land Hydrology.” In Biological Soil Crusts: An Organizing Principle in Drylands, edited by B. Weber , B. Büdel , and J. Belnap , 321–346. Cham: Springer International Publishing. 10.1007/978-3-319-30214-0_17.

[emi70033-bib-0008] Cheng, S. , W. Xian , Y. Fu , et al. 2019. “Genomes of Subaerial Zygnematophyceae Provide Insights Into Land Plant Evolution.” Cell 179, no. 5: 1057–1067.e14. 10.1016/j.cell.2019.10.019. 31730849

[emi70033-bib-0009] Colica, G. , H. Li , F. Rossi , D. Li , Y. Liu , and R. de Philippis . 2014. “Microbial Secreted Exopolysaccharides Affect the Hydrological Behavior of Induced Biological Soil Crusts in Desert Sandy Soils.” Soil Biology and Biochemistry 68: 62–70. 10.1016/j.soilbio.2013.09.017.

[emi70033-bib-0010] Dadras, A. , J. M. R. Fürst‐Jansen , T. Darienko , et al. 2023. “Environmental Gradients Reveal Stress Hubs Pre‐Dating Plant Terrestrialization.” Nature Plants 9, no. 9: 1419–1438. 10.1038/s41477-023-01491-0. 37640935 PMC10505561

[emi70033-bib-0011] De Vries, J. , and J. M. Archibald . 2018. “Plant Evolution: Landmarks on the Path to Terrestrial Life.” New Phytologist 217, no. 4: 1428–1434. 10.1111/nph.14975. 29318635

[emi70033-bib-0012] Domozych, D. S. , B. Wells , and P. J. Shaw . 1991. “Basket Scales of the Green Alga, Mesostigma Viride: Chemistry and Ultrastructure.” Journal of Cell Science 100, no. 2: 397–407. 10.1242/jcs.100.2.397.

[emi70033-bib-0013] Donner, A. , K. Glaser , N. Borchhardt , and U. Karsten . 2017. “Ecophysiological Response on Dehydration and Temperature in Terrestrial Klebsormidium (Streptophyta) Isolated From Biological Soil Crusts in Central European Grasslands and Forests.” Microbial Ecology 73, no. 4: 850–864. 10.1007/s00248-016-0917-3. 28011994

[emi70033-bib-0014] Duncan, T. M. , K. S. Renzaglia , and D. J. Garbary . 1997. “Ultrastructure and Phylogeny of the Spermatozoid of *Chara vulgaris* (Charophyceae).” Plant Systematics and Evolution 204, no. 3–4: 125–140. 10.1007/BF00989201.

[emi70033-bib-0015] Geraldes, V. , and E. Pinto . 2021. “Mycosporine‐Like Amino Acids (MAAs): Biology, Chemistry and Identification Features.” Pharmaceuticals 14, no. 1: 63. 10.3390/ph14010063. 33466685 PMC7828830

[emi70033-bib-0016] Glaser, K. , S. Kammann , N. Plag , and M. Dressler . 2023. “Ecophysiological Performance of Terrestrial Diatoms Isolated From Biocrusts of Coastal Sand Dunes.” Frontiers in Microbiology 14: 1279151. 10.3389/fmicb.2023.1279151. 38169811 PMC10758497

[emi70033-bib-0017] Glaser, K. , A. T. van , E. Pushkareva , I. Barrantes , and U. Karsten . 2022. “Microbial Communities in Biocrusts Are Recruited From the Neighboring Sand at Coastal Dunes Along the Baltic Sea.” Frontiers in Microbiology 13: 859447. 10.3389/fmicb.2022.859447. 35783389 PMC9245595

[emi70033-bib-0018] Glaser, K. , A. Donner , M. Albrecht , T. Mikhailyuk , and U. karsten . 2017. “Habitat‐Specific Composition of Morphotypes With Low Genetic Diversity in the Green Algal Genus Klebsormidium (Streptophyta) Isolated From Biological Soil Crusts in Central European Grasslands and Forests.” European Journal of Phycology 52, no. 2: 188–199. 10.1080/09670262.2016.1235730.

[emi70033-bib-0019] Glass, S. E. , R. M. McCourt , S. D. Gottschalk , L. A. Lewis , and K. G. Karol . 2023. “Chloroplast Genome Evolution and Phylogeny of the Early‐Diverging Charophycean Green Algae With a Focus on the *Klebsormidiophyceae* and *Streptofilum* .” Journal of Phycology 59, no. 6: 1133–1146. 10.1111/jpy.13359. 37548118

[emi70033-bib-0020] Harholt, J. , Ø. Moestrup , and P. Ulvskov . 2016. “Why Plants Were Terrestrial From the Beginning.” Trends in Plant Science 21, no. 2: 96–101. 10.1016/j.tplants.2015.11.010. 26706443

[emi70033-bib-0021] Hartmann, A. , K. Glaser , A. Holzinger , M. Ganzera , and U. Karsten . 2020. “Klebsormidin A and B, Two New UV‐Sunscreen Compounds in Green Microalgal Interfilum and Klebsormidium Species (Streptophyta) From Terrestrial Habitats.” Frontiers in Microbiology 11: 499. 10.3389/fmicb.2020.00499. 32292396 PMC7118736

[emi70033-bib-0022] Herburger, K. , and A. Holzinger . 2015. “Localization and Quantification of Callose in the Streptophyte Green Algae Zygnema and Klebsormidium: Correlation With Desiccation Tolerance.” Plant and Cell Physiology 56: pcv139. 10.1093/pcp/pcv139. PMC4650865 26412780

[emi70033-bib-0023] Herburger, K. , U. Karsten , and A. Holzinger . 2016. “Entransia and Hormidiella, Sister Lineages of Klebsormidium (Streptophyta), respond Differently to Light, Temperature, and Desiccation Stress.” Protoplasma 253, no. 5: 1309–1323. 10.1007/s00709-015-0889-z. 26439247 PMC4710678

[emi70033-bib-0024] van den Hoek, C. , D. Mann , and H. M. Jahns . 1996. Algae: An Introduction to Phycology. Cambridge, UK: Cambridge University Press.

[emi70033-bib-0025] Holzinger, A. , and U. Karsten . 2013. “‘Desiccation Stress and Tolerance in Green Algae: Consequences for Ultrastructure, Physiological and Molecular Mechanisms.” Frontiers in Plant Science 4: 327. 10.3389/fpls.2013.00327. 23986769 PMC3749462

[emi70033-bib-0026] Holzinger, A. , M. Y. Roleda , and C. Lütz . 2009. “The Vegetative Arctic Freshwater Green Alga Zygnema Is Insensitive to Experimental UV Exposure.” Micron 40, no. 8: 831–838. 10.1016/j.micron.2009.06.008. 19660959

[emi70033-bib-0027] Hotter, V. , K. Glaser , A. Hartmann , M. Ganzera , and U. Karsten . 2018. “Polyols and UV‐Sunscreens in the Prasiola‐Clade (Trebouxiophyceae, Chlorophyta) as Metabolites for Stress Response and Chemotaxonomy.” Journal of Phycology 54, no. 2: 264–274. 10.1111/jpy.12619. 29345725 PMC5947255

[emi70033-bib-0028] Irisarri, I. , T. Darienko , T. Pröschold , J. M. R. Fürst‐Jansen , M. Jamy , and J. de Vries . 2021. “Unexpected Cryptic Species Among Streptophyte Algae Most Distant to Land Plants.” Proceedings of the Royal Society B: Biological Sciences 288, no. 1963: 20212168. 10.1098/rspb.2021.2168. PMC8611356 34814752

[emi70033-bib-0029] Karsten, U. , K. Herburger , and A. Holzinger . 2016. “Living in Biological Soil Crust Communities of African Deserts—Physiological Traits of Green Algal Klebsormidium Species (Streptophyta) to Cope With Desiccation, Light and Temperature Gradients.” Journal of Plant Physiology 194: 2–12. 10.1016/j.jplph.2015.09.002. 26422081 PMC4710676

[emi70033-bib-0030] Karsten, U. , I. Klimant , and G. Holst . 1996. “A New In Vivo Fluorimetric Technique to Measure Growth of Adhering Phototrophic Microorganisms.” Applied and Environmental Microbiology 62, no. 1: 237–243. 10.1128/aem.62.1.237-243.1996. 16535211 PMC1388753

[emi70033-bib-0031] Kern, R. , V. Hotter , A. Frossard , et al. 2019. “Comparative Vegetation Survey With Focus on Cryptogamic Covers in the High Arctic Along Two Differing Catenas.” Polar Biology 42, no. 11: 2131–2145. 10.1007/s00300-019-02588-z.

[emi70033-bib-0032] Khanipour Roshan, S. , K. Dumack , M. Bonkowski , U. Karsten , and K. Glaser . 2020. “Stramenopiles and Cercozoa Dominate the Heterotrophic Protist Community of Biological Soil Crusts Irrespective of Edaphic Factors.” Pedobiologia 83: 150673. 10.1016/j.pedobi.2020.150673.

[emi70033-bib-0033] Kitzing, C. , T. Pröschold , and U. Karsten . 2014. “UV‐Induced Effects on Growth, Photosynthetic Performance and Sunscreen Contents in Different Populations of the Green Alga Klebsormidium Fluitans (Streptophyta) From Alpine Soil Crusts.” Microbial Ecology 67, no. 2: 327–340. 10.1007/s00248-013-0317-x. 24233286

[emi70033-bib-0034] Maden, A. R. , K. S. Renzaglia , and D. P. Whittier . 1996. “Ultrastructure of the Spermatozoid of *Lycopodium obscurum* (Lycopodiaceae).” American Journal of Botany 83, no. 4: 419–429. 10.1002/j.1537-2197.1996.tb12723.x.

[emi70033-bib-0035] Mikhailyuk, T. , A. Lukešová , K. Glaser , et al. 2018. “New Taxa of Streptophyte Algae (Streptophyta) From Terrestrial Habitats Revealed Using an Integrative Approach.” Protist 169, no. 3: 406–431. 10.1016/j.protis.2018.03.002. 29860113 PMC6071840

[emi70033-bib-0036] Mikhailyuk, T. I. , H. J. Sluiman , A. Massalski , et al. 2008. “New Streptophyte Green Algae From Terrestrial Habitats and an Assessment of the Genus Interfilum (Klebsormidiophyceae, Streptophyta).” Journal of Phycology 44, no. 6: 1586–1603. 10.1111/j.1529-8817.2008.00606.x. 27039871

[emi70033-bib-0037] Moestrup, Ø. 1970. “The Fine Structure of Mature Spermatozoids of *Chara corallina* , With Special Reference to Microtubules and Scales.” Planta 93, no. 4: 295–308. 10.1007/BF00384103. 24496766

[emi70033-bib-0038] Pedersen, Å. Ø. , P. Convey , K. K. Newsham , et al. 2022. “Five Decades of Terrestrial and Freshwater Research at Ny‐Ålesund, Svalbard.” Polar Research 41: 6310. 10.33265/polar.v41.6310.

[emi70033-bib-0039] Pierangelini, M. , K. Glaser , T. Mikhailyuk , U. Karsten , and A. Holzinger . 2019. “Light and Dehydration but Not Temperature Drive Photosynthetic Adaptations of Basal Streptophytes (*Hormidiella*, *Streptosarcina* and *Streptofilum*) Living in Terrestrial Habitats.” Microbial Ecology 77, no. 2: 380–393. 10.1007/s00248-018-1225-x. 29974184 PMC6394494

[emi70033-bib-0040] Pierangelini, M. , D. Ryšánek , I. Lang , W. Adlassnig , and A. Holzinger . 2017. “Terrestrial Adaptation of Green Algae Klebsormidium and Zygnema (Charophyta) Involves Diversity in Photosynthetic Traits but Not in CO_2_ Acquisition.” Planta 246, no. 5: 971–986. 10.1007/s00425-017-2741-5. 28721563 PMC5633629

[emi70033-bib-0041] Prelle, L. R. , A. Graiff , S. Gründling‐Pfaff , V. Sommer , K. Kuriyama , and U. Karsten . 2019. “Photosynthesis and Respiration of Baltic Sea Benthic Diatoms to Changing Environmental Conditions and Growth Responses of Selected Species as Affected by an Adjacent Peatland (Hütelmoor).” Frontiers in Microbiology 10: 1500. 10.3389/fmicb.2019.01500. 31333612 PMC6620715

[emi70033-bib-0042] R Development Core Team . 2022. R: A Language and Environment for Statistical Computing. Vienna, Austria: R Foundation for Statistical Computing.

[emi70033-bib-0043] Renzaglia, K. S. , T. H. Johnson , H. D. Gates , and D. P. Whittier . 2001. “Architecture of the Sperm Cell of Psilotum.” American Journal of Botany 88, no. 7: 1151–1163. 10.2307/3558326. 11454615

[emi70033-bib-0044] Ritchie, R. J. 2006. “Consistent Sets of Spectrophotometric Chlorophyll Equations for Acetone, Methanol and Ethanol Solvents.” Photosynthesis Research 89, no. 1: 27–41. 10.1007/s11120-006-9065-9. 16763878

[emi70033-bib-0045] Rogers, C. E. , K. R. Mattox , and K. D. Stewart . 1980. “The Zoospore of *Chlorokybus Atmophyticus*, a Charophyte With Sarcinoid Growth Habit.” American Journal of Botany 67, no. 5: 774–783.

[emi70033-bib-0046] Ronquist, F. , and J. P. Huelsenbeck . 2003. “MrBayes 3: Bayesian Phylogenetic Inference Under Mixed Models.” Bioinformatics 19, no. 12: 1572–1574. 10.1093/bioinformatics/btg180. 12912839

[emi70033-bib-0047] Samolov, E. , K. Baumann , B. Büdel , et al. 2020. “Biodiversity of Algae and Cyanobacteria in Biological Soil Crusts Collected Along a Climatic Gradient in Chile Using an Integrative Approach.” Microorganisms 8, no. 7: 1047. 10.3390/microorganisms8071047. 32674483 PMC7409284

[emi70033-bib-0048] Samolov, E. , T. Mikhailyuk , A. Lukešová , K. Glaser , B. Büdel , and U. Karsten . 2019. “Usual Alga From Unusual Habitats: Biodiversity of Klebsormidium (Klebsormidiophyceae, Streptophyta) From the Phylogenetic Superclade G Isolated From Biological Soil Crusts.” Molecular Phylogenetics and Evolution 133: 236–255. 10.1016/j.ympev.2018.12.018. 30576758

[emi70033-bib-0049] Sluiman, H. J. , C. Guihal , and O. Mudimu . 2008. “Assessing Phylogenetic Affinities and Species Delimitations in Klebsormidiales (Streptophyta): Nuclear‐Encoded rDNA Phylogenies and ITS Secondary Structure Models in Klebsormidium, Hormidiella and Entransia.” Journal of Phycology 44, no. 1: 183–195. 10.1111/j.1529-8817.2007.00442.x. 27041055

[emi70033-bib-0050] Starr, R. C. , and J. A. Zeikus . 1993. “UTEX—The Culture Collection of Algae at the University of Texas at Austin; 1993—List of Cultures.” Journal of Phycology 29, no. s2: 1–106. 10.1111/j.0022-3646.1993.00001.x.

[emi70033-bib-0051] Teoh, M.‐L. , S.‐M. Phang , and W.‐L. Chu . 2013. “Response of Antarctic, Temperate, and Tropical Microalgae to Temperature Stress.” Journal of Applied Phycology 25, no. 1: 285–297. 10.1007/s10811-012-9863-8.

[emi70033-bib-0052] Walsby, A. E. 1997. “Numerical Integration of Phytoplankton Photosynthesis Through Time and Depth in a Water Column.” New Phytologist 136, no. 2: 189–209. 10.1046/j.1469-8137.1997.00736.x.

[emi70033-bib-0053] Yan, W. , and L. A. Hunt . 1999. “An Equation for Modelling the Temperature Response of Plants Using Only the Cardinal Temperatures.” Annals of Botany 84, no. 5: 607–614. 10.1006/anbo.1999.0955.

[emi70033-bib-0054] Žárský, V. , and M. Eliáš . 2024. “Phylogenomics Defines *Streptofilum* as a Novel Deep Branch of Streptophyte Algae.” bioRxiv, 2024.03.08.584070. 10.1101/2024.03.08.584070. 40068608

[emi70033-bib-0055] Zuker, M. 2003. “Mfold Web Server for Nucleic Acid Folding and Hybridization Prediction.” Nucleic Acids Research 31, no. 13: 3406–3415. 10.1093/nar/gkg595. 12824337 PMC169194

