# Supplementary figures and images for: New Strains of the Deep Branching Streptophyte Streptofilum: Phylogenetic Position, Cell Biological and Ecophysiological Traits, and Description of Streptofilum arcticum sp. nov
Source: Environ Microbiol. 2025 Jan 8;27(1):e70033. doi: 10.1111/1462-2920.70033 (PMC11741916; doi:10.1111/1462-2920.70033)

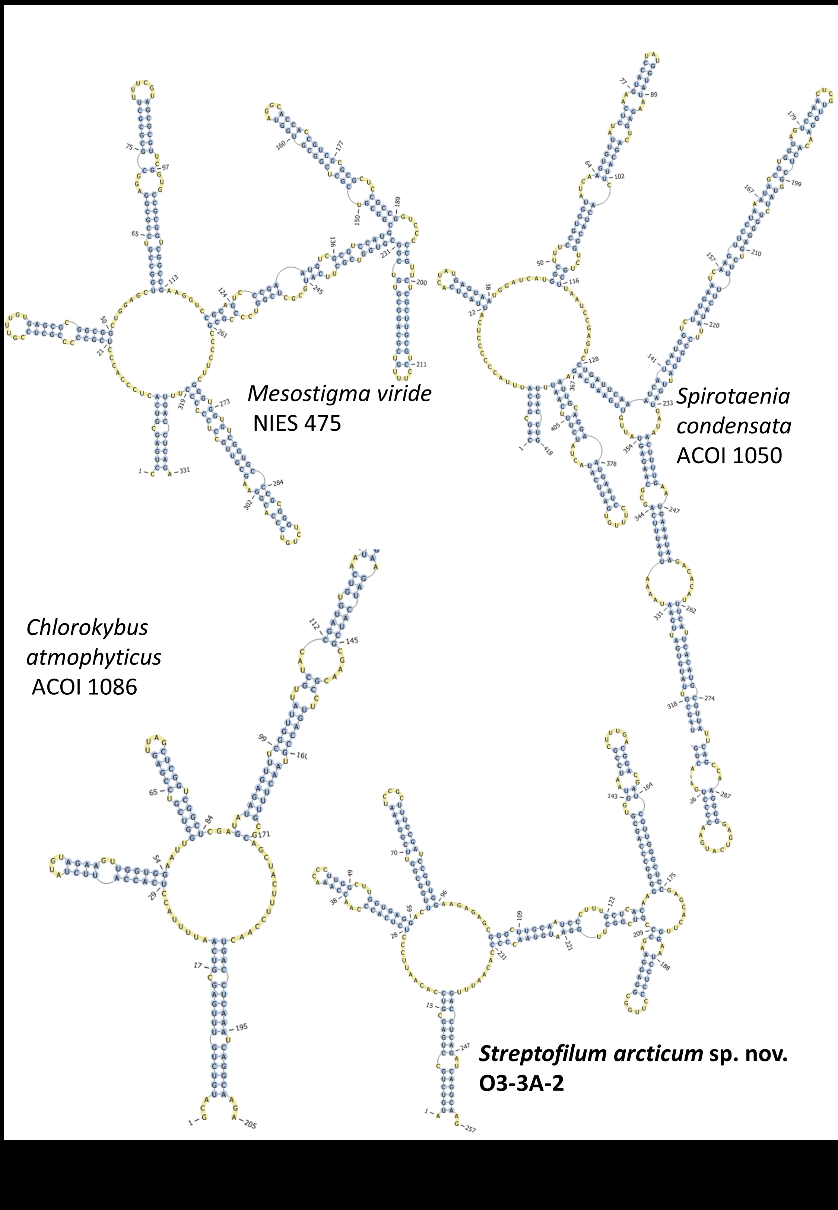
Supplementary fig. 1

Supplementary figure 2


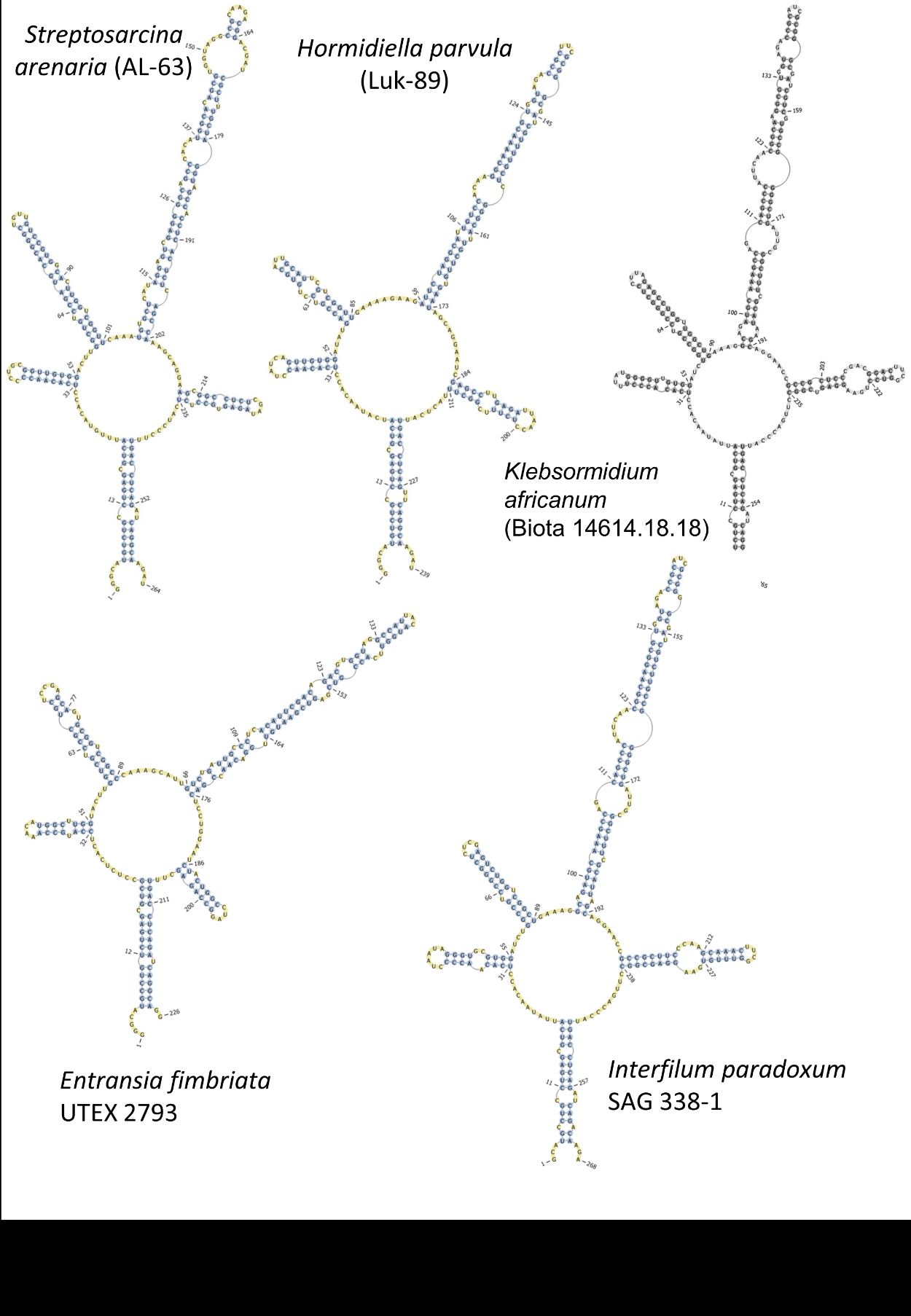


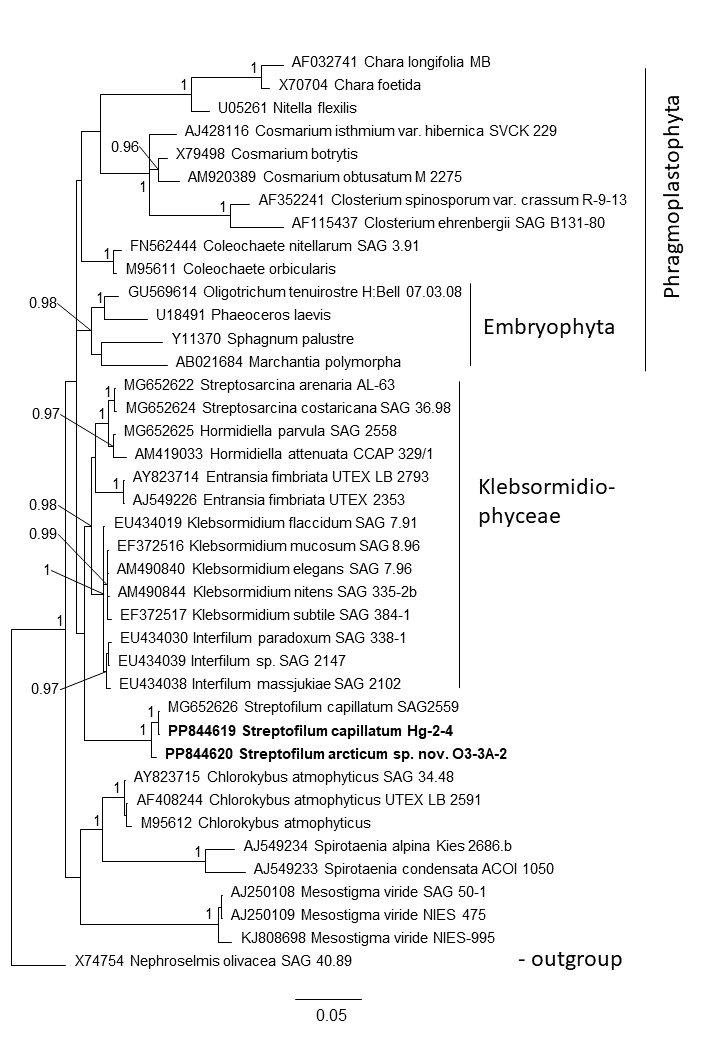


Supplementary figure 3


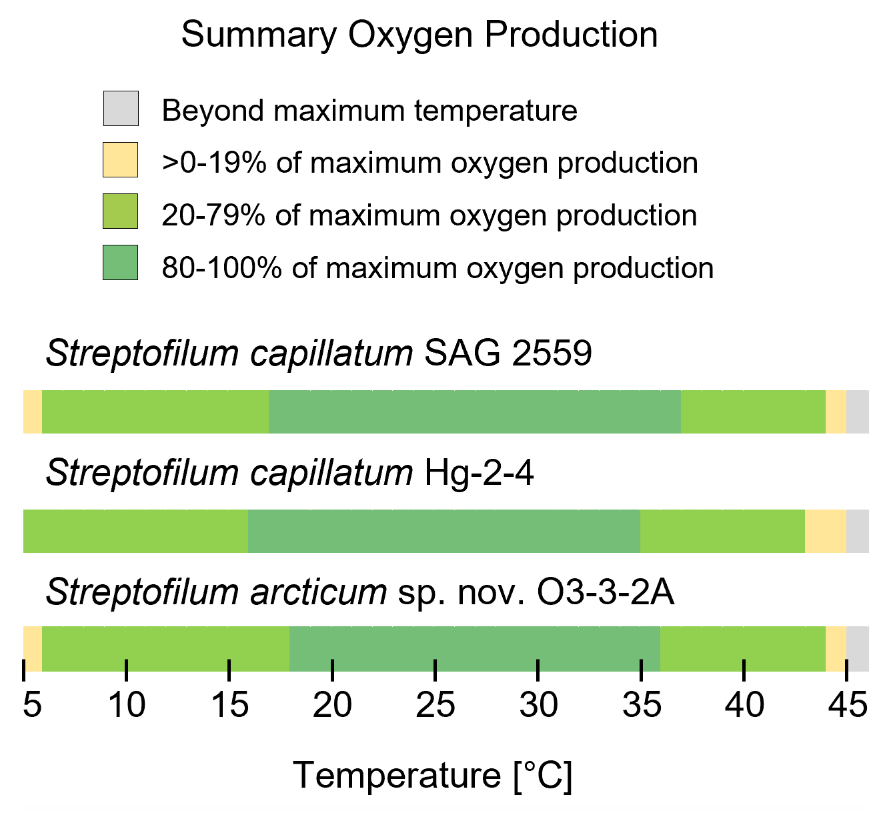


Supplementary figure 4

Supplement: Supplementary file 1 — Figure S1. ITS2 secondary structures of various Streptophyta, representing different phylogenetic lineages and Streptofilum. Figure S2. ITS2 secondary structures of different representatives of Klebsormidiophyceae (Streptophyta). Figure S3. Molecular phylogeny of Streptophyta based on SSU sequence comparisons. A phylogenetic tree was inferred by Bayesian method (program MrBayes) with Bayesian Posterior Probabilities (PP) indicated at nodes; values lower than 0.95 are not shown. Strain in bold represents newly sequenced Streptofilum strains. Figure S4. Optimum and maximum temperatures based on short‐term oxygen production of measurements and long‐term (few days, measured as growth‐rate) effects of temperature treatment on three Streptofilum strains (n = 4). Values were calculated based on the fitting results presented in Figure 8. [file EMI-27-e70033-s001.docx]
